# MicroRNA-665 and its potential role in drug response and survival outcomes in multiple myeloma: a preliminary study

**DOI:** 10.3389/fphar.2025.1465814

**Published:** 2025-04-04

**Authors:** Rui Bergantim, Sara Peixoto da Silva, Vanessa Pinto, Joana M. Pereira, Diana Sousa, Fernanda Trigo, Rune Matthiesen, José E. Guimarães, M. Helena Vasconcelos

**Affiliations:** ^1^ i3S – Instituto de Investigação e Inovação em Saúde, University of Porto, Porto, Portugal; ^2^ IPATIMUP - Institute of Molecular Pathology and Immunology of the University of Porto, Porto, Portugal; ^3^ Clinical Hematology, Hospital Center of São João, Porto, Portugal; ^4^ Clinical Hematology, FMUP – Faculty of Medicine of the University of Porto, Porto, Portugal; ^5^ Department of Biological Sciences, FFUP - Faculty of Pharmacy of the University of Porto, Porto, Portugal; ^6^ Faculty of Dental Medicine (FMD), Universidade Católica Portuguesa, Viseu, Portugal; ^7^ Unit for Multidisciplinary Research in Biomedicine (UMIB), School of Medicine and Biomedical Sciences (ICBAS), University of Porto, Porto, Portugal; ^8^ Laboratory of Integrative and Translocation Research in Population Health (ITR), Porto, Portugal; ^9^ Computational and Experimental Biology Group, iNOVA4Health, NOVA Medical School, Faculdade de Ciências Médicas, NMS|FCM, Universidade Nova de Lisboa, Lisboa, Portugal; ^10^ Instituto Universitário de Ciências da Saúde, Cooperativa de Ensino Superior Politécnico e Universitário IUCSESPU, Paredes, Portugal

**Keywords:** miRNAs, multiple myeloma, biomarkers, drug resistance, drug response

## Abstract

**Background:**

Multiple myeloma (MM) is a complex hematological malignancy with heterogeneous clinical and pathophysiological backgrounds that influence treatment responses and outcomes. Identifying biomarkers to predict drug response and guide treatment decisions, particularly regarding drug combinations, is essential to improve therapeutic efficacy and patient outcomes. This study explores the role of microRNAs (miRNAs/miRs) derived from bone marrow (BM) and peripheral blood (PB) in responses to treatment and survival outcomes in newly diagnosed MM (ndMM) patients.

**Methods:**

This study included twenty patients with ndMM undergoing first-line treatment with bortezomib, thalidomide, and dexamethasone. The miRNAs were isolated from BM and PB, and their profiles were analyzed using Next-Generation Sequencing (NGS), followed by validation of differentially expressed miRNAs by quantitative real-time PCR (qPCR). Clinical and response data were collected to assess correlations between miRNA levels, clinical characteristics, and patient outcomes. *In silico* analysis for target-prediction and gene ontology (GO) enrichment was performed to explore the potential biological and functional role of the identified miRNAs.

**Results:**

NGS profiling revealed several miRNAs differently expressed between treatment-refractory and sensitive patients, as well as between PB and BM. Among these, miR-665, miR-483-5p, miR-143-3p and miR-145-5p were selected for further validation by qPCR. It was observed that miR-665 was significantly elevated in treatment-refractory patients compared to treatment-sensitive patients. Additionally, miR-665 levels were higher in PB than in BM. Elevated miR-665 levels were associated with more aggressive disease characteristics and poorer clinical outcomes, including reduced overall survival.

**Discussion:**

Our preliminary findings suggest that miR-665 could potentially serve as a non-invasive tool for predicting drug resistance and guiding treatment decisions in MM. These findings also highlight the potential utility of miRNAs in liquid biopsies as a predictive tool of drug response in MM and could pave the way for personalized treatment strategies, improving patient outcomes. Future research is needed to validate these results in larger cohorts and explore the underlying mechanisms of miR-665 in MM pathogenesis and drug resistance.

## Introduction

Multiple myeloma (MM) is a highly clinical, phenotypical, and molecular heterogeneous hematological neoplasia, making the perfect treatment approach challenging to establish. Over the last few years, the MM treatment landscape has significantly changed with new drugs such as proteasome inhibitors (PIs), immunomodulators (IMiDs), and several monoclonal antibodies approved and broadly used to treat MM. Additionally, other therapies with new mechanisms of action, such as conjugated and bi-specific monoclonal antibodies and Chimeric antigen receptor (CAR) T-cells, have been integrated into clinical practice, allowing new treatment perspectives (Rodriguez-Otero et al., 2021; Gulla and Anderson, 2020). The impact of these treatments, both at diagnosis and relapses, has been substantial, enabling MM patients to achieve better responses, longer progression-free survival (PFS), and ultimately better overall survival (OS) (Thorsteinsdottir et al., 2018). This is particularly evident in the first line of treatment, which usually includes a PI, an IMiD, and, recently, an anti-CD38 monoclonal antibody. These improvements have led to higher rates of complete response and undetectable measurable residual disease (MRD) in patients either eligible or non-eligible for transplant (Diamond et al., 2021; Munshi et al., 2020).

Nonetheless, a small percentage of patients fail to respond to initial therapy and are classified as primary refractory MM patients (Jurczyszyn et al., 2020; Liu et al., 2021; Majithia et al., 2015). It has been reported that around 10%–20% of MM patients are refractory to first-line treatments, including PIs and IMiDs, with around 5% being refractory to recent anti-CD38^+^ monoclonal antibodies-based protocols (Jurczyszyn et al., 2020; Lahuerta et al., 2021). There is no consensus on the management of these patients, making them a particularly complex group to treat, exhibiting very aggressive disease characteristics and markedly shorter survival outcomes (Liu et al., 2021; Majithia et al., 2015).

The current treatment algorithm includes first-line therapy with multiple drug combinations, intensified or not with autologous stem cell transplant. Subsequent lines of treatment are determined according to the mechanisms of action, the response achieved, and the toxicity of previous therapies (Dimopoulos et al., 2021). At each step of the treatment, patient characteristics (such as performance status) and disease characteristics (including cytogenetic abnormalities) are evaluated to contribute to informed therapeutic decisions (Gengenbach et al., 2021; Hernández-Rivas et al., 2022; Bonello et al., 2022). However, these approaches fail to identify primary resistant patients and different outcomes are achieved by patients treated with the same therapeutic protocol (Dimopoulos et al., 2021; Moreau et al., 2021). Thus, biomarkers to predict drug response and guide treatment decisions, particularly in selecting optimal drug combinations, are essential to improve therapeutic efficacy and outcomes in MM.

MicroRNAs (miRNAs/miRs) are highly conserved, small non-coding RNAs (approximately 19–25 nucleotides long) that modulate gene expression at both transcriptional and post-transcriptional levels (Lin and Gregory, 2015). miRNAs specifically bind (fully or partially) to complementary regions within target mRNAs to promote their degradation and/or inhibit their translation, depending on the degree of complementarity (Ha and Kim, 2014). By degrading or blocking the translation of mRNA targets, miRNAs regulate gene expression in cancer pathophysiology, such as genes involved in several cellular signaling pathways, apoptosis, cell growth, proliferation, invasion, and metastasis (Pedroza-Torres et al., 2019; Suzuki et al., 2015; Sousa et al., 2020). By targeting oncogenes and tumor suppressor genes, miRNAs are major players in the hallmarks of cancer and drug response (Zhang et al., 2007; Giovannetti et al., 2012; Peixoto da Silva et al., 2021). In MM, aberrant miRNA expression has been reported, with numerous upregulated or downregulated miRNAs identified across different processes of tumorigenesis (Bergantim et al., 2024; Pinto et al., 2020). For instance, several studies demonstrated that specific miRNAs may negatively influence the drug sensitivity of MM cells at different stages of the disease, emphasizing their possible role in developing drug resistance (Handa et al., 2019; Abdi et al., 2016; Allegra et al., 2021a; Campo et al., 2014; Chen et al., 2021; Ismail et al., 2023).

Considering the biological and clinical heterogeneity observed in MM and the knowledge that miRNAs are regulators of various pathways contributing to the hallmarks of cancer, they have recently emerged as potential key biomarkers for diagnosing this disease (Zhang et al., 2007; Zhu et al., 2018; Xiang et al., 2021). Since miRNAs may be found in biological fluids such as blood (within circulating cells, associated with extracellular vesicles, or free) and can be easily detected, they represent promising candidates as non-invasive biomarkers in MM (Zhang et al., 2015; Rocci et al., 2014a; Rocci et al., 2014b; Krishnan and Bebawy, 2023; Bergantim et al., 2022). Identifying and quantifying free miRNAs circulating in the peripheral blood of MM patients may contribute to understanding drug resistance mechanisms and improve the prediction of treatment responses (Allegra et al., 2021b; Fang et al., 2023; Li et al., 2021; Zhang et al., 2016). In this study, we explore the role of miRNAs isolated from the bone marrow (BM) and peripheral blood (PB) of MM patients undergoing treatment with bortezomib, thalidomide, and dexamethasone to assess their potential impact on treatment response and survival outcomes.

## Material and methods

### Patients with MM and treatments

This study prospectively included twenty patients with newly diagnosed MM (ndMM), consecutively proposed for the first line of treatment with bortezomib, thalidomide, and dexamethasone at Centro Hospitalar and Universitário São João (CHUSJ) - Porto, Portugal, from January 2017 to January 2018. None of the patients had undergone any prior therapy before collecting PB and BM samples. Patients were classified according to their response to the treatment regimen into two groups: treatment-sensitive or treatment-primary refractory. Sensitive patients had a complete response (CR) after the second and fourth cycles of treatment that persisted with negative MRD at day 100 following autologous stem cell transplant. Primary refractory patients had no response after two cycles of the first-line treatment. For these primary refractory patients, additional therapeutic regimens were explored. The criteria for diagnosis, clinical staging, risk stratification, and treatment response were assessed according to the International Multiple Myeloma Group (IMWG) recommendations (Dimopoulos et al., 2021; Rajkumar et al., 2014; Kumar et al., 2016; Rajkumar et al., 2011). This study was approved by the Ethical Committee for Health at CHUSJ, and written informed consent was obtained from all participants in compliance with the Declaration of Helsinki.

### Collection and preparation of PB and BM samples

For all the twenty patients, PB and BM were collected. The 20 PB and 20 BM samples were simultaneously collected in Ethylenediaminetetraacetic acid (EDTA) tubes before and after treatment and processed within 1–4 h after collection. In addition, all samples were collected in the morning, in fasting patients, to avoid possible physiologic confounding factors. The samples were mixed with an equal volume of sterile phosphate saline buffer (PBS). Then, 4 mL of the patient samples (PB or BM) in PBS were carefully loaded over the top of Histopaque-1077 (Sigma-Aldrich, Steinheim, Germany) and placed in a 15 mL centrifuge tube. Then, the samples were centrifuged at 400 *g* (Centrifuge 5810R, Eppendorf), without brake, for 30 min at room temperature (RT). From this centrifugation, four layers resulted: a bottom layer containing erythrocytes and granulocytes, a second layer corresponding to the Histopaque, a third layer of mononuclear cells, and an upper layer corresponding to the cell-free plasma. This latter layer was then collected and aliquoted in several vials, immediately stored at −80°C to preserve RNA for further processing and analysis.

### miRNA extraction for library preparation

Volumes of approximately 1 mL of PB plasma and 1.5 mL of BM plasma were used to extract miRNAs. Before the extraction, and to increase the extraction yield, samples were treated with proteinase K (3 μg/μL) at 37°C for 10 min. Then, miRNA extraction was performed using the miRCURY™ RNA Isolation Kit (Exiqon, Foster City, CA, United States), following the manufacturer’s instructions. Before starting the lysis step, 10 μL of β-mercaptoethanol and 1 μg of MS2 RNA (RNA from bacteriophage MS2, Roche, Germany) were added to the lysis solution. The remaining protocol was performed as per the manufacturer’s instructions. To elute the RNA, 30 μL of RNase-free water was used. The RNA concentration of the samples was measured using a NanoDrop 1,000 Spectrophotometer (Thermo Scientific, Wilmington, DE, United States). All the purified RNA samples were stored at −80°C.

### Library preparation and miRNA profiling by next-generation sequencing (NGS)

miRNA cDNA libraries were constructed following the Ion Total RNA-Seq kit v2 protocol (Life Technologies, Carlsbad, CA, United States). After RNA quality control of samples, using the small RNA assay kit in the Bioanalyzer (Agilent 2,100, Santa Clara, CA, United States), 3.5 μL of total RNA from each PB and BM sample was incubated for 16 h to facilitate adaptors hybridization and ligation. After incubation, reverse transcription was performed, and cDNA was purified and size-selected by magnetic beads. Afterwards, cDNA was amplified with a specific primer with unique barcodes to identify and track each sample. Due to the presence of an adapter dimer in some samples, an adapted protocol was developed where final libraries run in agarose gel and bands were excised, avoiding the adapter dimer band. Then, the pooled libraries were processed using the Ion Chef™ System (Life Technologies, Carlsbad, CA, United States), and the resulting 550™ chips were sequenced on the Ion S5™XL System (Cheng and Hill, 2017).

### Bioinformatics

A total of 35 FASTQ files from the Ion Torrent sequencer were analyzed. The files underwent trim processing using CutAdapt with the following settings: a minimum fragment length of 10 base pairs and a minimum quality score of 20 were specified (Schmieder and Edwards, 2011). Following the trimming process using CutAdapt, the quality of the resulting reads was evaluated with FASTQC to ensure acceptable quality standards (FastQC. Available online: http://www.bioinformatics.babraham.ac.uk/projects/fastqc, accessed on 20 December 2019). Reads were then aligned by Bowtie1 to human genome assembly (hg19), allowing for one mismatch. Prebuild indexes were downloaded from the Bowtie webpage. The length of seed substrings in the bowtie analysis was set to 10, and the additional parameters—best–nomaqround were also specified. The read count was calculated using FeatureCounts (Liao et al., 2014) software implemented in miARma-Seq (Andrés-León and Rojas, 2019) using a minimum quality of 10. The genome annotation files were miRBase_Annotation_20_for_hsa_mature_miRNA.gtf and/Homo_sapiens.GRCh37.75. gtf for miRNAs and all RNAs, respectively. Significant differential expression was calculated using the EdgeR package (Robinson et al., 2010), with a minimum count per million cut-off of 2. biomaRt (Durinck et al., 2009) was used to retrieve transcript annotation. To generate visualizations for our data analysis, we utilized the ggplot2 R package to create Venn diagrams, Pie charts, and Volcano plots.

### Candidate miRNA confirmation by qPCR

miRNAs found differentially expressed between PB and BM samples and differentially expressed between samples from refractory and sensitive patients were selected for further validation of the NGS results by quantitative real-time PCR (qPCR). RNA from those samples was extracted using miRNeasy Serum/Plasma Advanced Kit (217204, Qiagen, Germany), following the manufacturer’s instructions. To increase the recovery, 1 μg of MS2 RNA (RNA from bacteriophage MS2, Roche, Germany) was added to the RPL Buffer on each sample, as recommended by the manufacturer. For each sample, 400 μL were used as input, which resulted in a 30 μL final elution volume. Total RNA concentration and purity were measured with a NanoDrop 1,000 Spectrophotometer (Thermo Scientific, Wilmington, DE, United States). Total RNA (10 ng) served as a template for cDNA synthesis using the TaqMan^TM^ Advanced miRNA cDNA Synthesis Kit (Applied Biosystems, Foster City, CA, United States), according to the manufacturer’s protocol and using the T100 Thermal Cycler (Bio-Rad, Hercules, CA, United States). The miRNAs expression levels were assessed using 2X TaqMan™Fast Advanced Master Mix (Applied Biosystems, Foster City, CA, United States) and 20X TaqMan™ Advanced miRNA Assays probes (hsa-miR-145-5p, 477916_mir; hsa-miR-143-3p, 477912_mir; hsa-miR-483-5p, 478432_mir; hsa-miR-665, 479150_mir–Applied Biosystems, Foster City, CA, United States), according to the manufacturer’s instructions (in a final volume of 20 μL per well). The qPCR reactions were performed in a 7500 Fast Real-Time PCR device (Applied Biosystems, Foster City, CA, United States). For each sample, triplicates were performed, and negative controls lacking cDNA template (no template control) or lacking reverse transcription enzyme (no reverse transcription) were included in duplicate in the qPCR plates. Three miRNAs found by NGS to be abundantly expressed in all samples were tested as endogenous controls (hsa-miR-26a-5p, 477995_mir; hsa-miR-17-5p, 478447_mir; hsa-miR-103a-3p, 478253_mir–Applied Biosystems, Foster City, CA, United States). The miR-26a-5p, already reported as a reference to normalize miRNA qPCR levels in hematological malignancies (Damanti et al., 2021), was then selected for expression normalization since its expression was the most stable in all samples (when compared to the other two candidates by using the BestKeeper software (Pfaffl et al., 2004) and the analysis of cycle threshold (Ct) values among the samples - Supplementary Figure S1). Additionally, RefFinder was used to analyze and confirm the potential use of miR-26a-5p as endogenous control (Xie et al., 2023).

The efficiency of all the qPCR assays was between 90% and 110%, as recommended by the manufacturer. The Ct values for all miRNAs in each sample were acquired with the 7500 Software v2.06 (Applied Biosystems, Foster City, CA, United States). The Livak Method (2^−ΔΔCT^), along with the Student’s t-test (when data followed normality) or with the Mann-Whitney U test (when data did not follow normality), were used to determine statistical differences in the normalized relative expression of miRNAs among the different groups.

### Statistical analysis

The clinical characteristics of primary refractory patients to treatment were compared with those sensitive to treatment, along with the differential levels of the miRNAs of interest. Boxplots were employed to visualize the miRNA expression levels across the groups. Outliers were identified using the interquartile range (IQR) method and included in the analysis and the *p*-value calculations. Retaining the outliers was deemed appropriate, as they likely represent natural biological variability. Nominal variables were expressed as percentages and analyzed using the Chi-square test, with proper corrections applied when needed, such as the Yates correction for continuity or Fisher’s exact test. For continuous variables, differences between the two groups were evaluated using a two-sided independent Student's t-test when the data were normally distributed and with the Mann-Whitney U test when the assumption of normality was not met or the variable was ordinal. To determine the cut-off for the identified miRNAs, ΔCT values were utilized to reduce technical variability and ensure robust miRNA expression measurements. Subsequently, Youden’s Index was applied to these values to identify the optimal cut-off, sensitivity, and specificity for each miRNA (Yang et al., 2022). Analyses of overall survival (OS) and progression-free survival (PFS) were conducted. OS was defined by the duration from the start of the treatment of disease to death (regardless of the cause of death), and PFS was defined by the duration from the start of the treatment to disease progression or death (regardless of the cause of death), whichever comes first (Rajkumar et al., 2011). Kaplan–Meier curves for OS and PFS were constructed, and differences were assessed using the Mantel-Cox (log-rank) test. Univariate COX regression analyses were performed for exploratory purposes to identify potential predictors. Variables found to be statistically significant in the univariate analysis were subsequently included in multivariate Cox regression models to identify independent predictors of outcomes. Furthermore, a penalized Cox regression model using Lasso regularization was applied to address potential multicollinearity and overfitting in the context of a small sample size. Hazard ratios (HRs) with 95% confidence intervals (CIs) were calculated for all analyses (Abd ElHafeez et al., 2021). Results were considered statistically significant when *p*-values were ≤0.05. Statistical analyses were conducted using IBM SPSS Statistics for Macintosh (Version 29.0.2.0, IBM Corp., Armonk, NY, United States) (Corp, 2023) and GraphPad Prism (version 10.0.0 for Mac, GraphPad Software, Boston, Massachusetts United States) (Prism, 2024).

### *In silico* gene ontology analysis

miR-665 gene targets were predicted using an ensemble approach composed of three different target prediction bioinformatic tools (miRDB, TargetScanHuman 8.0, and DIANA TOOLS—microT-CDS) (Chen and Wang, 2020; McGeary et al., 2019; Paraskevopoulou et al., 2013). To minimize prediction errors, only gene targets with a miRDB Target Score ≥80, TargetScan cumulative weighted context score ≤ − 0.3, and DIANA micro-T-CDS prediction score ≥0.8 were selected. Genes identified by at least two different prediction tools were used for enrichment analysis using Enrichr (Kuleshov et al., 2016). All Gene Ontology (GO) terms with a q-value ≤0.05 and a combined enrichment score over 10 were considered. GOnet was used to visualize relationships between miR-665 target genes and statistically significant GO terms (Pomaznoy et al., 2018).

## Results

### Baseline clinical characteristics of MM patients

A cohort of twenty newly diagnosed patients with MM was included in the study. The median age at diagnosis was 58 years old (ranging from 44 to 67), with 60% of male patients. At diagnosis, the predominant clinical feature was anemia (80%), followed by lytic lesions (60%), renal failure (30%) and hypercalcemia (25%). Additionally, 15% of patients presented extramedullary disease, primarily non-paraskeletal. Regarding the revised international staging system (R-ISS), three patients (15%) were R-ISS stage I, eleven (55%) were R-ISS stage II and six (30%) were R-ISS stage III. Cytogenetics by Fluorescence *In Situ* Hybridization (FISH) revealed high-risk cytogenetics abnormalities in 20% of the patients, with two of them exhibiting double-hit features, including the deletion 17p and t(4;14)(p16;q32). In terms of treatment response, eight patients achieved a CR with the induction treatment and maintained CR after autologous stem cell transplant, while twelve were classified as primary refractory. Of note, abnormal FISH results (*p* = 0.017) and high lactate dehydrogenase (LDH) levels (*p* = 0.035) were statistically significantly different between sensitive and primary refractory patients. Additionally, a trend suggested R-ISS stage III to be more related to primary refractory patients (*p* = 0.053). A detailed summary of the baseline clinical characteristics of the MM patients is provided in Table 1.

**TABLE 1:** Baseline and clinical characteristics of Multiple Myeloma (MM) patients according to their treatment response.

Baseline and clinical characteristics	Total (n = 20)	Refractory (n = 12)	Sensitive (n = 8)	p-value
Age				
Median (range) - yr	58 (44–67)	56 (47–63)	63 (44–67)	*0.163*
Sex - no. (%)				
Male	12 (60%)	9 (75%)	3 (37.5%)	*0.094*
Female	8 (40%)	3 (25%)	5 (62.5%)	
Myeloma isotype - no (%)				
Light Chain Kappa	4 (20%)	2 (16.7%)	2 (25%)	*0.247*
IgG/k	6 (30%)	2 (16.7%)	4 (50%)	
IgG/L	5 (25%)	5 (41.7%)	0 (0%)	
IgA/k	2 (10%)	2 (16.7%)	1 (12.5%)	
IgA/L	3 (15%)	1 (8.3%)	1 (12.5%)	
ISS stage - no (%)				
I	3 (15%)	1 (8.3%)	2 (25%)	*0.300*
II	8 (40%)	4 (33.3%)	4 (50%)	
III	9 (45%)	7 (58.3%)	2 (25%)	
R-ISS stage - no (%)				
I	3 (15%)	1 (8.3%)	2 (25%)	*0.053*
II	11 (55%)	5 (41.7%)	6 (75)	
III	6 (30%)	6 (50%)	0 (0%)	
FISH				
Normal	11 (55%)	4 (33.3%)	7 (87.5%)	0.017*
Abnormal	9 (45%)	8 (66.7%)	1 (12.5%)	
High-risk cytogenetic profile				
No	5 (25%)	4 (33.3%)	1 (12.5%)	*0.134*
Yes	4 (20%)	4 (33.3%)	0 (0%)	
High-risk cytogenetic profile				
del (17p)	2 (10%)	2 (22.2%)	0 (0%)	*0.571*
t (4:14)	2 (10%)	2 (22.2%)	0 (0%)	*0.572*
t (14; 16)	1 (5%)	1 (8.3%)	0 (0%)	*0.708*
t (11; 14)	3 (15%)	2 (16.7%)	1 (12.5%)	*0.134*
amp1q21	3 (15%)	3 (15%)	0 (0%)	*0.453*
Double-Hit (del17p and t (4; 14))	2 (10%)	2 (16.7%)	0 (0%)	*0.571*
Anemia				
<10 g/dL or <1 g/dL of normal Hb	16 (80%)	11 (91.6%)	5 (62.5%)	*0.110*
Creatinine				
<2 mg/dL	14 (70%)	7 (58.3%)	7 (87.5%)	*0.163*
≥2 mg/dL	6 (30%)	5 (41.7%)	1 (12.5%)	
Hipercalcemia				
yes	5 (25%)	4 (33.3%)	1 (12.5%)	*0.292*
Lytic lesions				
yes	12 (60%)	6 (50%)	6 (75)	*0.264*
B2-microglobulin				
Increased	9 (45%)	7 (58.3%)	2 (25%)	*0.142*
Normal	11 (55%)	5 (41.7%)	6 (75)	
LDH				
Increased	5 (25%)	5 (41.7%)	0 (0%)	0.035*
Normal	15 (75%)	7 (58.3%)	8 (100%)	
Extramedullary plasmacytoma				
Yes	3 (15%)	3 (25%)	0 (0%)	*0.125*
No	17 (85%)	9 (75%)	8 (100%)	
Clonal plasma cell in BM (median, range)	20.9 (5%–72%)	26.5 (5%–72%)	16.4 (12%–64%)	*0.702*
Clonal plasma cell in PB (median, range)	0 (0.35%–4%)	0.58 (0.35%–4%)	0 (0%–0%)	*0.089*

BM, bone marrow; FISH, fluorescence *in situ* hybridization; ISS, international staging system; LDH, lactate dehydrogenase; PB, peripheral blood; R-ISS, revised international staging system.

*p ≤ 0.05.

### miRNA profiling by NGS

**FIGURE 1:**
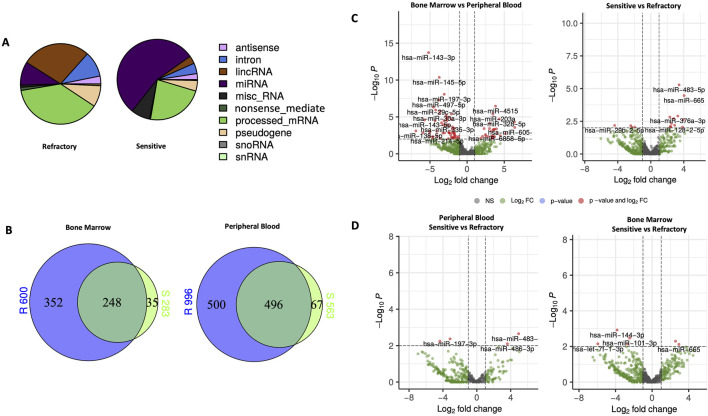
Characteristics and expression of circulating miRNAs in bone marrow (BM) and peripheral blood (PB) of primary refractory and sensitive Multiple Myeloma (MM) patients using NGS analysis. **(A)** Composition of the mapped reads of circulating RNAs in primary refractory and responsive MM patients combining BM and PB. **(B)** Venn diagram of significantly differentially expressed miRNAs according to response status (refractory vs sensitive) and type of sample (BM vs PB); **(C)** Volcano plots of the results of the differential miRNAs expression analyses according to response status (refractory vs sensitive) and type of sample (BM vs PB); **(D)** Volcano plots of the results of differential miRNAs expression analysis according to response status (refractory vs sensitive) in both PB and BM. The miRNAs with a |log2 fold change| ≥1.5 and FDR *p*-value < 0.05 are labeled in red in the plots. miRNA, microRNA; lincRNA, long intergenic noncoding RNA; miscRNA, miscellaneous other RNA; snoRNA, small nucleolar RNA; snRNA, small nuclear RNA.

To characterize the small RNAs, sequencing was performed on samples from both BM and PB of the twenty patients included in this study. The distribution patterns of the mapped reads revealed distinct RNA compositions between sensitive and primary refractory patients, as illustrated in the pie chart in Figure 1A. In sensitive patients, a significant majority (55%) of mappable RNAs were miRNAs, whereas in primary refractory patients, miRNAs represented only 15% of the reads. The remaining RNA types in both groups were attributed to small nuclear or nucleolar RNA, long intergenic noncoding RNA, and miscellaneous small RNA. Notably, in primary refractory patients, most mapped reads corresponded to processed mRNA (60%).

Venn diagrams (Figure 1B) were used to compare the differences between sensitive and primary refractory patients regarding the expression of miRNAs in PB and BM samples. This analysis identified 653 miRNAs in BM, of which 600 were in primary refractory patients and 283 in sensitive patients, and 1,063 miRNAs in PB, of which 996 were in primary refractory patients and 563 were in sensitive patients. Furthermore, this analysis showed an overlap of 248 miRNAs in the BM and 496 miRNAs in the PB when comparing the expression profiles of primary refractory *versus* sensitive patients.

To identify differentially expressed miRNAs with statistical significance, volcano plots (Figure 1C) were generated, applying filtering criteria for both PB and BM samples, as well as distinguishing between sensitive and refractory patients. Furthermore, volcano plots were created to illustrate the differences between sensitive and refractory patients in both PB and BM (Figure 1D). The applied threshold used to screen up- or downregulated miRNAs was a log2 fold change ≥1.5 and False Discovery Rate (FDR) *p-value* ≤ 0.05. In the plots, the red points represent the differentially expressed miRNAs with statistical significance. Table 2 summarizes the top ten upregulated and downregulated miRNAs in refractory patients compared to sensitive patients and the most significantly upregulated and downregulated miRNAs in PB compared to BM.

**TABLE 2:** miRNAs based on their NGS expression patterns (upregulated or downregulated) in refractory patients and peripheral blood.

	miRNA	logFC	logCPM	*p-value*
UPREGULATED IN REFRACTORY PATIENTS	hsa-miR-483-5p	3,430800011	7,839680282	5,18E-06
	hsa-miR-665	4,039370272	5,932134772	3,42E-05
	hsa-miR-376a-3p	3,272721022	6,099817551	0,001257833
	hsa-miR-4667-5p	3,933875948	5,304446229	0,002388779
	hsa-miR-3176	3,841951429	5,468643693	0,008870548
	hsa-miR-187-5p	4,467481157	4,999912736	0,014689868
	hsa-miR-5001-3p	4,11926043	5,037955191	0,026838139
	hsa-miR-873-3p	3,252375639	4,839430741	0,032849171
	hsa-miR-520g-3p	3,52583699	4,858377697	0,044649489
	hsa-miR-4722-5p	3,217018137	4,872146192	0,047432659
DOWNREGULATED IN REFRACTORY PATIENTS	hsa-miR-29b-2-5p	−4,379510925	6,573692081	0,006573483
	hsa-miR-95-3p	−3,910144848	6,013312209	0,02079349
	hsa-let-7f-1-3p	−4,848958739	5,735267299	0,023131113
	hsa-miR-1246	−4,430617075	6,174884833	0,023838552
	hsa-miR-361-3p	−3,465796319	5,992564948	0,02388589
	hsa-miR-877-3p	−3,306627765	5,898643163	0,034312164
	hsa-miR-338-3p	−4,479214903	6,276779402	0,038914405
	hsa-miR-616-5p	−4,134781364	5,353101873	0,039685056
	hsa-miR-330-5p	−4,484324636	6,227873871	0,041484991
	hsa-miR-33a-3p	−4,487403017	5,565536783	0,048197106
UPREGULATED IN PERIPHERAL BLOOD	hsa-miR-4515	3,821164973	7,354851532	3,47E-07
	hsa-miR-328-5p	4,314346645	6,469368173	1,92E-05
	hsa-miR-4269	6,354529751	6,110361793	4,96E-05
	hsa-miR-605-5p	6,407563131	6,004300064	0,000267493
	hsa-miR-4669	3,949008303	6,415231537	0,000417561
	hsa-miR-6858-5p	4,011935719	5,530086494	0,001843167
	hsa-miR-586	5,146168885	5,39453953	0,001897352
	hsa-miR-4505	4,470723829	5,332474608	0,003524915
	hsa-miR-1304-3p	3,707346522	5,731713067	0,004909534
	hsa-miR-583	4,462684862	4,89663433	0,041073489
DONWREGULATED IN PERIPHERAL BLOOD	hsa-miR-143-3p	−5,201131981	12,24935304	1,89E-14
	hsa-miR-145-5p	−3,752119667	12,73229925	4,32E-11
	hsa-miR-143-5p	−5,734877574	7,014021433	2,59E-05
	hsa-miR-138-5p	−6,901637807	7,171435152	0,000785347
	hsa-miR-218-5p	−5,093952615	7,252905501	0,0008404
	hsa-miR-5571-3p	−5,955884531	6,457745261	0,001385308
	hsa-miR-642a-3p	−5,032561789	5,897212976	0,001532273
	hsa-miR-511-5p	−5,012709257	5,857941789	0,004520526
	hsa-miR-1275	−5,47465861	6,156047374	0,013981566
	hsa-miR-582-5p	−4,924638241	5,825759013	0,016247235

logCPM, log count-per-million; LogFC, log Fold Change; miRNA, microRNA.

For validation purposes, specific miRNAs were selected based on their differential expression. miR-483-5p and miR-665, both upregulated in primary refractory patients compared to the sensitive ones, were chosen for further analysis. Additionally, miR-143-3p and miR-145-5p, both upregulated in BM compared to PB samples, were also selected for subsequent validation studies. The selection for miRNAs further analysis was based on several criteria: the higher log Fold Change (logFC) reflecting the most significant expression level variations, the smallest *p-*values suggesting that the observed difference in expression is unlikely to have occurred by chance, and those with the lowest FDR indicating a higher confidence in the result being a true positive rather than a false discovery.

### Candidate miRNA confirmation by qPCR

#### miR-665 and miR-483-5p levels are higher in primary refractory patients compared to sensitive patients

**FIGURE 2:**
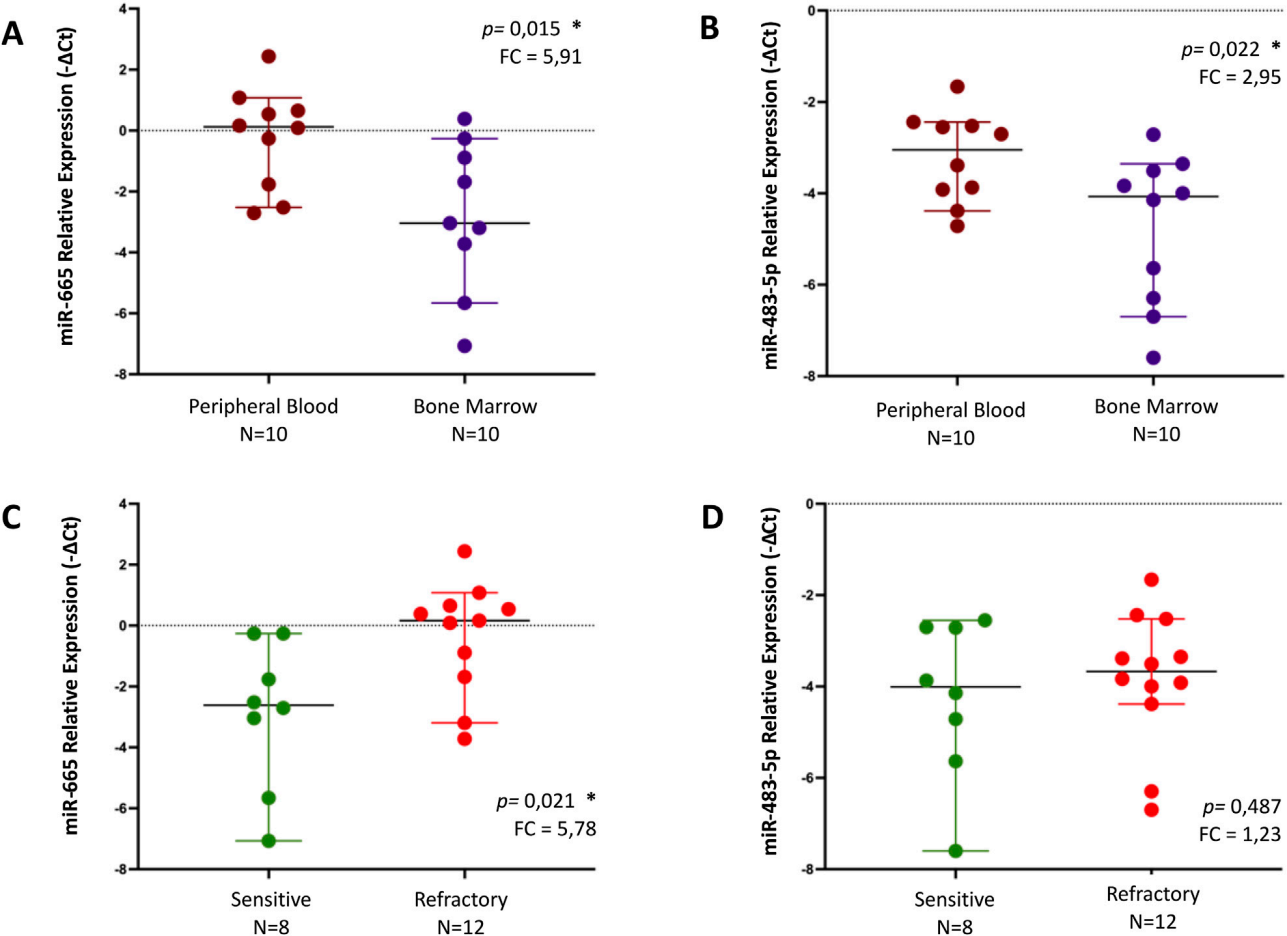
Relative expression of miR-655 and miR-483-5p, by qPCR, in different samples - peripheral blood (PB) vs bone marrow (BM) - **(A, B)** and regarding response to treatment (sensitive vs refractory) in MM patients **(C, D)**. Differences in miRNA expression levels between groups were analyzed using t-test. Results are presented in dot plots that present the median with a 95% Confidence Interval. FC: Fold Change. *p ≤ 0.05.

It was observed that miR-665 was significantly more abundant in the PB (n = 10) than in the BM (n = 10), with a fold change of 5.91 (*p* = 0.015) (Figure 2A). The same trend was observed for miR-483-5p, with a fold change of 2.95 (*p* = 0.022) (Figure 2B). Additionally, a statistically significant increase was observed in the levels of miR-665 in primary refractory patients' samples (n = 12) when compared to sensitive patients' samples (n = 8), with a fold of 5.78 (*p* = 0.021) (Figure 2C). There was also an increase in the levels of miR-483-5p in primary refractory patients' samples (n = 12) compared to sensitive patients' samples (n = 8), although without statistical significance, with a fold change of 1.23 (*p* = 0.487) (Figure 2D). All differences in miRNA expression levels between groups were analyzed using t-test.

#### miR-143-3p and mir-145-5p levels are higher in BM when compared to PB

**FIGURE 3:**
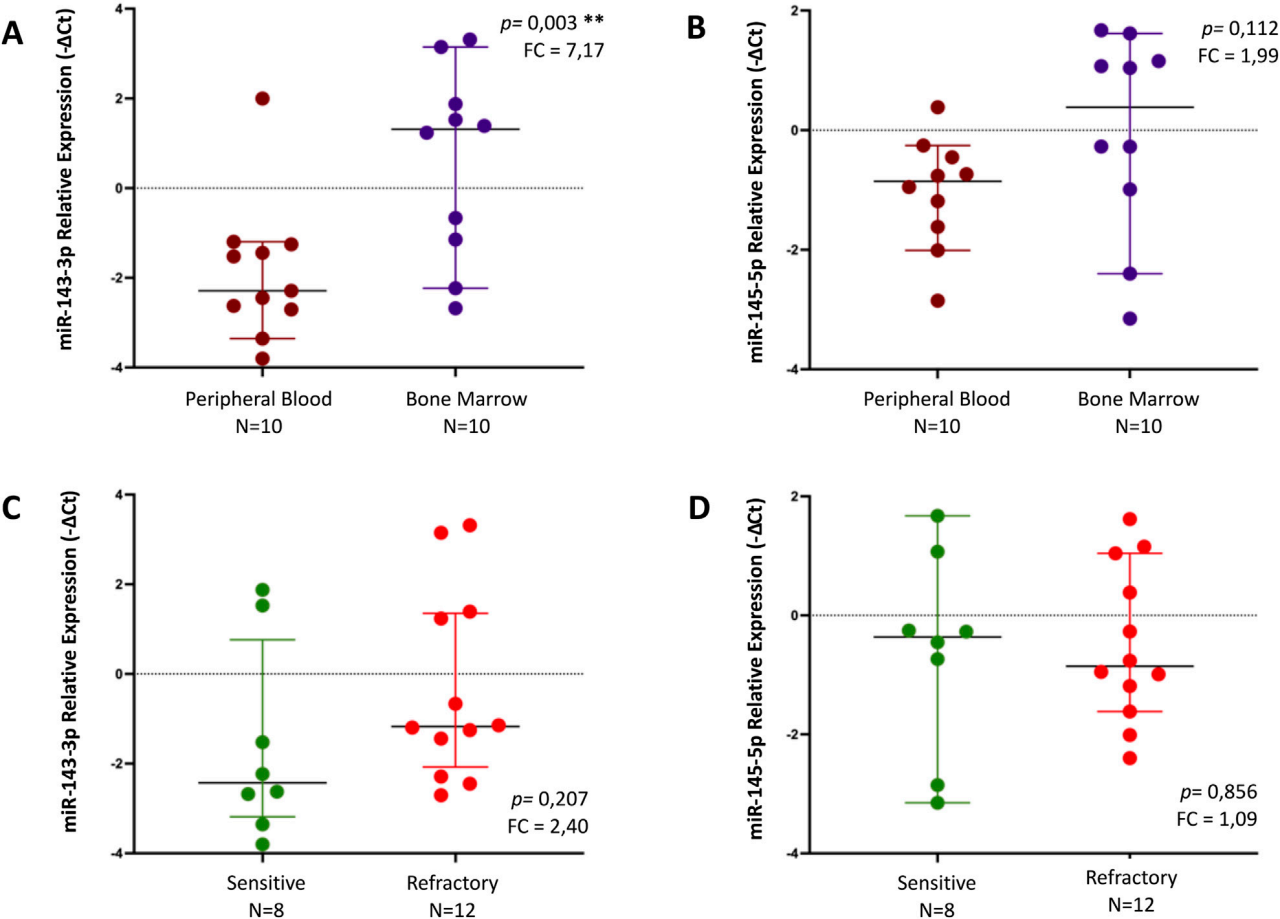
Relative expression of miR-143-3p and miR-145-5p, by qPCR, in different samples - peripheral blood (PB) vs bone marrow (BM) - **(A, B)** and regarding response to treatment (sensitive vs refractory) in MM patients **(C, D)**. Differences in miRNA expression levels between groups were analyzed using t-test. Results are presented in dot plots that present the median with a 95% Confidence Interval. FC: Fold Change. *p ≤ 0.05. **p ≤ 0.01.

A statistically significant increase was observed in miR-143-3p levels in the BM (n = 10) compared to the PB samples (n = 10), with a fold change of 7.17 (*p* = 0.003) (Figure 3A). Regarding miR-145-5p, there was a trend toward increased levels in BM (n = 10) compared to those of PB (n = 10) with a fold change of 1.99, albeit not reaching statistical significance (*p* = 0.112) (Figure 3B). Neither miR-143-3p nor miR-145-5p showed statistically significant differences concerning treatment outcomes (Figures 3C, D). All differences in miRNA expression levels between groups were analyzed using t-test.

#### Correlation of miRNAs expression with clinical characteristics and survival outcomes

To further assess the impact of miR-665 on clinical outcomes, survival analysis was performed using the Kaplan-Meyer curves and Cox regression analysis. To assess a cut-off value for miR-665 expression, a receiver operating characteristics (ROC) curve analysis was performed using the previously defined complete response to treatment as the dependent variable. This analysis revealed an area under the curve of 0.83, with a 95% confidence interval ranging from 0.651 to 1.000 and a *p*-value of 0.014 (Supplementary Figure S2). Youden’s index confirmed the optimal cut-off for miR-665, identified as ΔCT 0.2206, yielding a sensitivity of 100% and a specificity of 33%. For further analysis, the miRNA-665 was dichotomized based on low (ΔCT <0.2206, n = 10) *versus* high expression (ΔCT ≥0.2206, n = 10).

#### miR-665 is associated with aggressive clinical characteristics

All the samples were collected before treatment, underscoring the possibility of finding differences that could serve as biomarkers. Given its higher expression in primary refractory patients and detectability in both BM and PB, miR-665 was selected to evaluate whether its expression is associated with known clinical characteristics. Notably, levels of miR-665 were associated with advanced stages of the R-ISS and aggressive clinical features (Supplementary Table S1). In more detail, a statistical significance was found between increased expression of miR-665 and elevated creatinine (*p* = 0.010, Fisher’s exact test), elevated b2-microglobulin (*p* = 0.028 Fisher’s exact test), elevated LDH (*p* = 0.035, Fisher’s exact test), the presence of extramedullary disease (*p* = 0.021, Fisher’s exact test), and the stage III R-ISS (*p* = 0.03, Fisher’s exact test). However, high-risk cytogenetics features were not associated with elevated miR-665 levels (*p* = 0.343, Fisher’s exact test).

#### miR-665 is associated with poor survival outcomes

**FIGURE 4:**
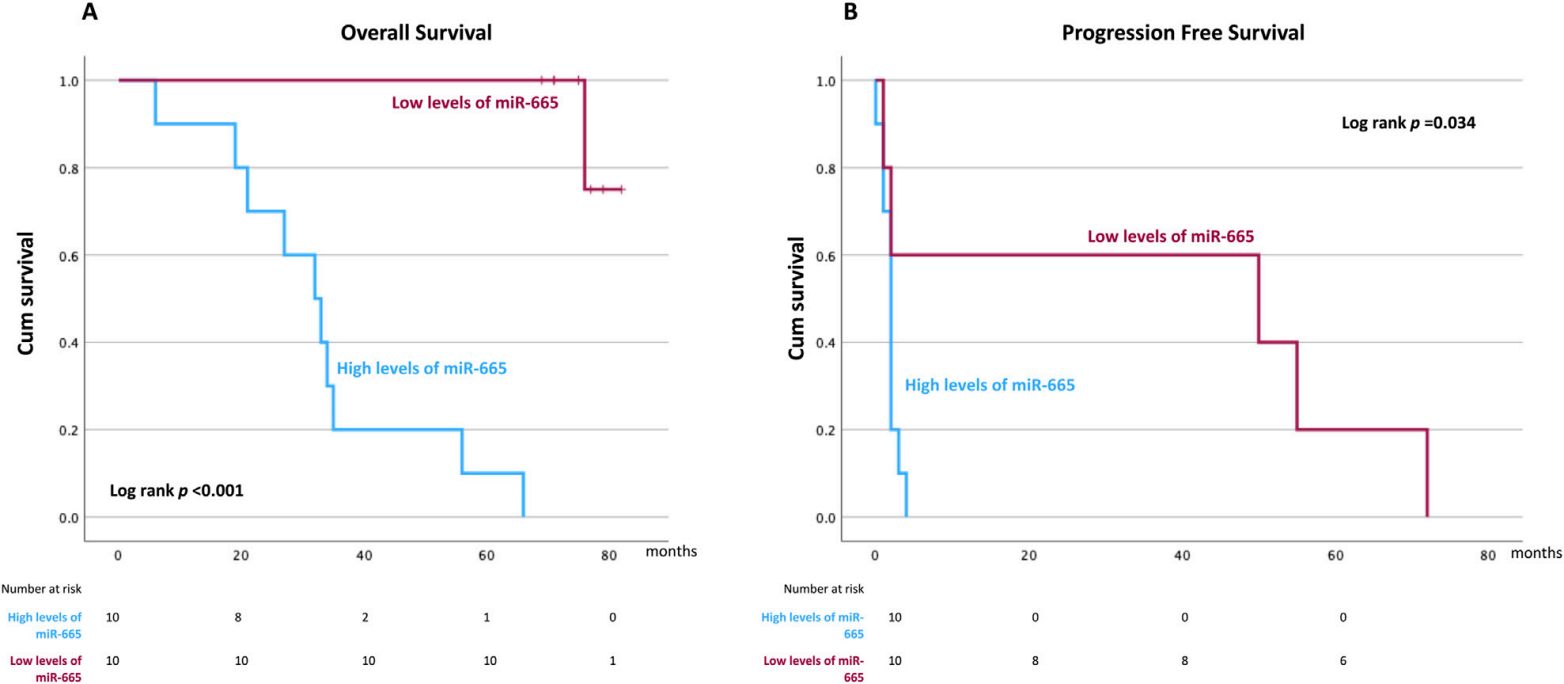
Kaplan-Meier curves illustrate the potential of miR-665 expression as a biomarker in peripheral blood (PB) for analyzing patient survival outcomes. The miRNA-665 was dichotomized based on low (ΔCT <0.2206) *versus* high expression (ΔCT ≥0.2206). **(A)** Overall Survival (OS). **(B)** Progression Free Survival (PFS).

The median follow-up for the entire cohort was of 75 months (range 6–82 months). Regarding OS, 11 events were identified, Kaplan-Meier survival analysis showing shorter OS in patients with higher levels of miR-665 (ΔCT ≥0.2206) in the PB, compared to those with lower levels (ΔCT <0.2206), with a median OS of 32 months for the first group and not reached for the second one (log-rank *p*-value < 0.001) (Figure 4A). Univariate Cox regression analysis demonstrated that MM patients levels of miR-665 impacted significantly OS (HR 1.787, 95% CI 1.145–2.789, *p* = 0.011), as well as R-ISS 3 (HR 4.624, 95% CI0.051-4.651, *p* = 0.021), increased LDH (HR 2.826, 95% CI 0.033-1.453, *p* = 0.047) and male gender (HR 0.171, 95% CI 0.132-6.425 *p* = 0.026). Nonetheless, multivariate Cox regression analysis did not show independent or retained significance in the penalized model used (Supplementary Table S2). Regarding PFS, with 15 events identified, the Kaplan-Meier survival analysis showed a shorter PFS in patients with higher levels of miR-665 compared to those with lower levels in the PB, with median PFS of 2 months and 50 months, respectively (log-rank *p*-value = 0.034) (Figure 4B). However, no variable showed independent significance in the univariate or multivariate Cox regression analysis (Supplementary Table S2).

#### *In silico* functional miRNA analysis

**FIGURE 5:**
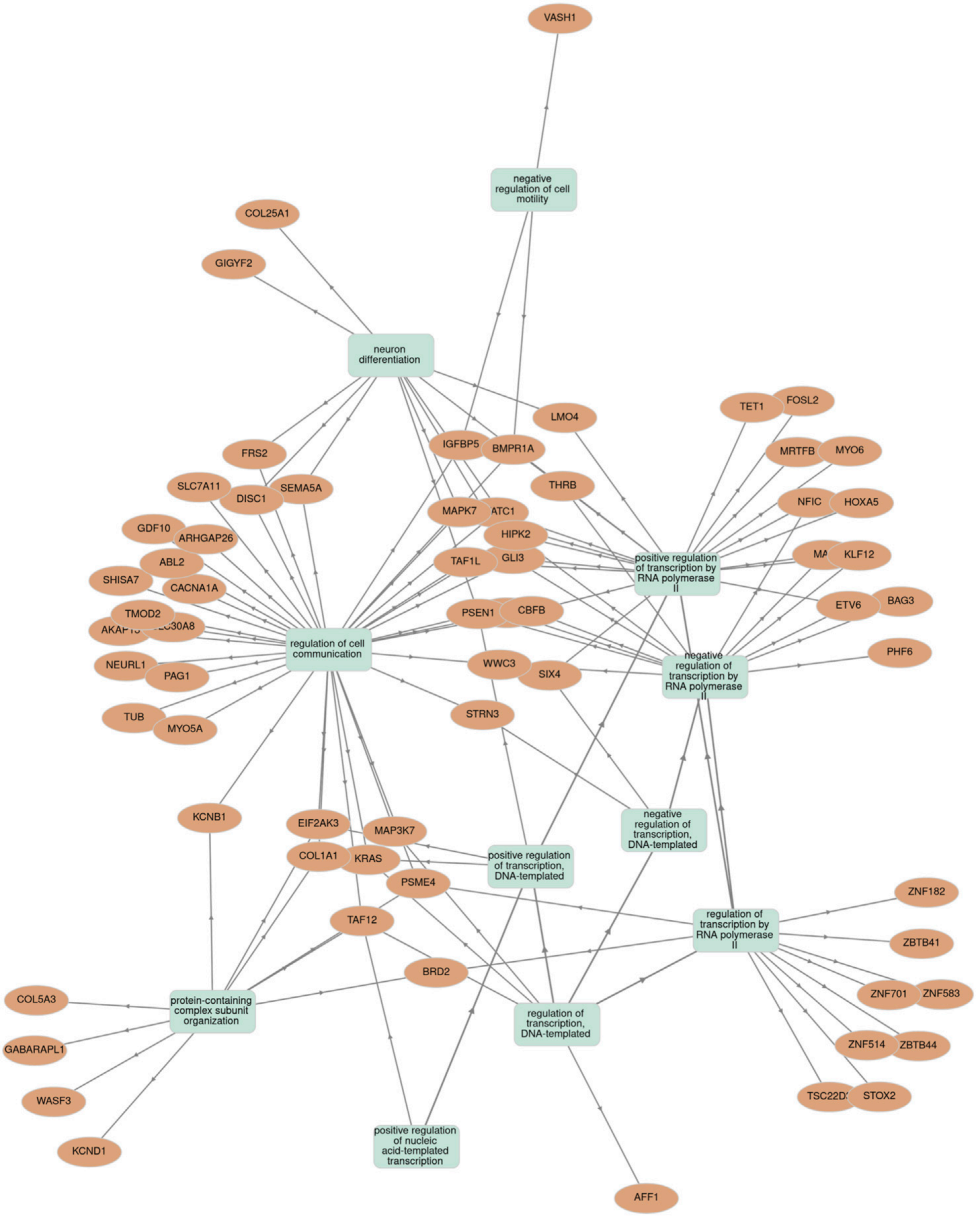
Association between miR‐665 target genes and statistically significant biological processes terms using the Gene Ontology (GO) enrichment tool.

To elucidate the possible biological and functional role of miR-665, an *in silico* analysis for target prediction and GO enrichment was performed. The potential target genes of miR-665 were assessed using miRDB, TargetScanHuman 8.9, and DIANA TOOLS-microT-CDS (Chen and Wang, 2020; McGeary et al., 2019; Paraskevopoulou et al., 2013). This comprehensive approach revealed 36 common genes across the databases and the main biological processes involved (Supplementary Figure S3A; Supplementary Figure S3B). Subsequent GO enrichment analysis showed that these genes are involved mainly in biological processes focusing on the regulation of DNA-templated transcription, regulation of transcription of RNA polymerase I and II, response to vascular endothelial growth factor (VEFG), cell migration and communication, and regulation of histone deacetylation (Supplementary Figure S3C). To further comprehend the functional roles of miR-665 and its expression, GO enrichment analysis was used to explore the correlation between the identified target genes and the statistically significant biological processes previously identified (Figure 5) (Kuleshov et al., 2016; Pomaznoy et al., 2018).

## Discussion

Treatment of newly diagnosed MM has changed substantially over the last years, with the incorporation of drugs from different classes featuring novel mechanisms of action. The current treatment algorithm includes first-line therapy with multiple drug combinations and several intensification and maintenance strategies based on the presence of high-risk cytogenetic abnormalities. This approach allows more intensive treatment of patients with these abnormalities, while those without such characteristics receive less intensive treatment. Nonetheless, there is a clear need for reliable biomarkers to guide therapeutic decisions more effectively. In this context, the study of biomarkers, such as miRNAs, for predicting treatment response is of foremost interest to enable a more personalized approach to MM treatment. The present study provides evidence that specific miRNAs are differentially expressed in newly diagnosed MM patients, their expression correlates with treatment response, and they can be assessed in BM and PB samples.

Using NGS, it was observed that sensitive and primary refractory patients exhibited different patterns in the RNA composition. In sensitive patients, the most mappable RNAs were miRNAs, whereas, in primary refractory patients, processed mRNAs were more common. This turned our attention to the fact that these differences in composition can indicate different disease characteristics and outcomes to be explored as biomarkers, such as miRNA abundance being correlated with treatment sensitivity or processed mRNA dominance in primary refractory patients, indicating the presence of resistance mechanisms. Moreover, different RNA degradation or stability patterns between groups could explain these differences (Sousa et al., 2020).

Notably, it was observed that miR-665 and miR-483-5p levels are elevated in primary refractory patients compared to sensitive patients. Additionally, despite having higher levels in BM samples, they were also detectable and quantified in PB samples. This variation in the expression levels of some miRNAs between groups of patients with different drug responses suggests their potential to be used as tools for treatment approaches. Therefore, these miRNAs were selected for further investigation in the current study. Several miRNAs are associated with drug resistance, such as miR-29b, associated with PIs resistance; miR-155, associated with daratumumab resistance; and miR-22, associated with IMiDs resistance (Caracciolo et al., 2021; Jagannathan et al., 2015; Azaman et al., 2023). The two differentially expressed miRNAs identified in our primary refractory patients, miR-665 and miR-483-5p, have been previously described to be involved in several biological processes and signaling pathways in hematological malignancies (Gu et al., 2022; Guan et al., 2023; Liu et al., 2019; Wang et al., 2022). However, there is a lack of evidence specifically addressing their role in MM drug resistance.

Previous studies have shown that miR-483-5p is located at the 11p15.5 locus of insulin-like growth factor (*IGF2*) gene and has been implicated in promoting cancer cell differentiation, invasion, metastasis, escape from apoptosis, drug resistance, and decreasing radiosensitivity in several solid cancers (Yang et al., 2017; Niture et al., 2023; Wang et al., 2018; Rattanapan et al., 2021; Tian et al., 2019). In MM, it was observed that plasmatic miR-483-5p was upregulated in MM patients compared to healthy controls and was associated with shorter median PFS, suggesting that miR-483-5p may serve as both a diagnostic and prognostic tool in MM (Qu et al., 2014). More recently, miR-483-5p has been identified as an oncomiR, promoting cell proliferation and inhibiting apoptosis in cell lines (Gu et al., 2022). Moreover, it was found that miR-483p-5p is upregulated in MM-mesenchymal stem cells (MM-MSCs) and could be transferred from these cells to MM cells via Extracellular Vesicles (EVs), favoring MM progression and related bone disease by targeting tissue inhibitor of metalloproteinase 2 (TIMP2) (Gu et al., 2022).

miR-665, whose gene is located at the 14q32.2 locus next to the immunoglobulin heavy locus (*IGH*) gene, essential to MM pathogenesis, has a complex and context-dependent function, once it plays diverse roles in different cancer types and can act either as an oncogene or as a tumor suppressor (Guan et al., 2023). For instance, it has been reported to be downregulated in osteosarcoma, retinoblastoma, and ovarian cancer, but upregulated in non-small cell lung cancer and breast cancer (Guan et al., 2023; Wu et al., 2020). In hematological malignancies, miR-665 appears to play a significant role in diffuse large B-cell lymphoma (DLBCL), acute lymphoblastic leukemia (ALL), and chronic myeloid leukemia (CML) (Guan et al., 2023). In DLBCL, expression of miR-665 can suppress tumor progression by targeting and inhibiting LIM and SH3 domain protein 1 (*LASP1*) and *MYC*, both essential for invasion, migration, and cell proliferation (Wang et al., 2022; Miao et al., 2019). In ALL, increased expression of miR-665 inhibits cell growth and promotes apoptosis through targeting exotoxin glycosyltransferase 1 (EXT1) and the extracellular signal-regulated kinase 1/2 (ERK1/2) signaling pathways, translating into better survivals. miR-665 also targets the transforming growth factor beta receptor 1 (*TGFBR1*) and the multidrug resistance-associated protein 2 (*ABCC2*), increasing drug efflux, reducing apoptosis, and promoting cycle arrest, proliferation, and drug resistance (Liu et al., 2019). In CML cells, the long non-coding RNA ADORA2A-AS1 upregulates TGFBR1 and ABCC2 expression by adsorbing miR-665, further promoting drug resistance (Guan et al., 2023; Liu et al., 2022). Regarding MM, the specific role and function of miR-665 remain unknown at the moment.

Although both miR-483-5p and miR-665 were found to be upregulated in primary refractory MM patients through NGS, further qPCR analysis showed that only the miR-665 retained statistical significance. This finding prompted us to explore its clinical implications in the analyzed patients. In addition to its association with drug response, our study highlights the potential of miR-665 as a prognostic tool in MM. Higher levels of miR-665 were associated with poorer OS, although it did not stand out as an independent factor in multivariate analysis. The same trend was observed for PFS, as expected once it had been observed that miR-665 was associated with a poor response to first-line treatment.

Furthermore, miR-665 was associated with an aggressive MM phenotype characterized by a higher disease burden, as evidenced by higher LDH, b2-microglobulin, and creatinine, as well as a higher incidence of extramedullary disease. Other studies associated higher levels of miRNAs with high-risk features, mainly extramedullary disease (Gregorova et al., 2019; Ahmad et al., 2014; Besse et al., 2015). For instance, miR-130a was found to be lower in the PB but higher in plasma cells from extramedullary tumors in comparison with BM plasma cells, suggesting that miR-130 could be taken up from circulation by the extramedullary tumor, thereby supporting angiogenesis and promoting cell growth and proliferation (Besse et al., 2015). However, in the present study, no samples were obtained from the extramedullary sites to confirm this association. When comparing with established prognostic models, higher levels of miR-665 were associated with a stage III of the R-ISS, indicating the potential utility of this miRNA as a biomarker or as an additional component to this prognostic staging tool, particularly to discriminate patients that are stage II. Curiously, the miR-665 gene is located next to the *IGH* gene in chromosome 14, which is essential in MM pathogenesis. Although higher levels of miR-665 were more common in patients with high cytogenetic risk, this difference did not reach statistical significance in the present study. Previous studies showed that, for example, that miR-133a was overexpressed in cases with t(14;16), a high-risk feature, showing a conserved inverse relation between several miRNAs deregulated in MM and cyclin D2 (CCND2) expression levels (Gutiérrez et al., 2010). Similarly, upregulated miR-99b or downregulated miR-744 have been associated with t(4;14), a major marker of poor prognosis in MM patients (Huang et al., 2012; Kubiczkova et al., 2014). Although miR-655 was not identified as an independent factor, it may reflect the complex MM landscape in these patients and could serve as a non-invasive prognostic tool for forecasting drug resistance and patient outcomes and refining the classical models currently available.

Concerning the differences between BM and PB samples, both miR-665 and miR-483-5p presented higher levels in PB compared to BM. Even though BM studies, including cytogenetics, remain the gold standard for diagnosis and monitoring, the potential to analyze circulating miRNAs in PB as liquid biopsies can represent a non-invasive tool. This could complement other biomarkers to follow-up treatment outcomes or be used alongside MRD.

Functional *in silico* analysis for miR-665 revealed that several target genes play essential roles in various biological processes and signaling pathways related to MM. Notably, genes such as *KRAS, BAG3, AFF1, CLI3, MAP3K7, WWC3*, and *HOXA5* are critical in regulating cell proliferation and apoptosis, and *TET1* plays a crucial role in epigenetic regulatio*n* (Pasca et al., 2019; Perroud et al., 2023; Bai and Chen, 2020; Harman et al., 2021; Egan et al., 2012; Liu et al., 2014; Carvajal-Vergara et al., 2005; Höffken et al., 2021; Huang et al., 2016; Muylaert et al., 2022). In the set of microenvironment modulation, *BAG3*, *TAF12,* and *MYO6* are important for facilitating the complex intercellular communication network (Bai and Chen, 2020; Ohata et al., 2016; Zub et al., 2015). Moreover, some of these genes, including *BAG3*, *BRD2*, *EIF2AK3*, *GABARAPL1*, *KRAS*, *PSME4, and SLC7A11,* have been implicated in modulating responses to drugs such as PIs and IMiDs (Zub et al., 2015; De Marco et al., 2020; Zuo and Liu, 2022; Leung-Hagesteijn et al., 2013; Sha et al., 2018; Yazgili et al., 2022; Zhang et al., 2023).

This functional analysis and the potential mechanisms mediated by miR-665 regulation highlight the heterogeneity of MM and the challenge in predicting treatment outcomes. Identifying specific miRNAs associated with treatment resistance or sensitivity would represent a significant step in the personalized approach of MM patients, addressing one of the most pressing unmet needs in the field. In this study, miR-665 appears to be a promising candidate for further investigation, given its association with higher-risk features typically related to treatment resistance, increased disease burden, and pooer treatment outcomes. However, a comprehensive approach to validate the direct interaction between miR-665 and the several potential targets is essential to truly understand the role of this miRNA in plasma cell proliferation and drug resistance mechanisms. This validation could be achieved by manipulating miRNA levels using synthetic miRNA mimics or miRNA inhibitors in cells, followed by functional assays to assess the effects on cellular behavior.

Furthermore, there are challenges and limitations associated with translating these findings into clinical practice. Firstly, there is a need for standardized protocols for miRNA detection and quantification, as variability in methods can lead to inconsistent results. Secondly, the functional relevance of miRNAs remains complex to determine, as they often have multiple targets, and their targets can be influenced by other miRNAs. Thus, further research is needed to clarify and consolidate the roles of miRNAs in MM. Thirdly, the small sample size of our study limits the generalization of the results and increases the influence of confounding factors such as environmental factors, concomitant treatments, genetic background, cytogenetic abnormalities, and mutations that modulate signaling pathways relevant to MM. More controlled and extensive studies are needed to validate miR-665 as an independent biomarker of drug resistance. These studies should include larger cohorts of patients, more comprehensive multivariate analyses, experimental validation of miRNA-target interactions, improved stratification of patient subgroups based on genetic risk, clinical profiles, and different treatment protocols. For instance, miR-665 analysis should be included in metanalyses along with other miRNAs and in prospective clinical trials to verify its potential as a possible biomarker for future use. Of particular interest would be the integration of miR-665 levels with other biomarkers and clinical features to enhance the current predictive tools available for precision medicine.

## Conclusion

The present study demonstrated that specific miRNAs are differently expressed between MM primary refractory and sensitive patients to first-line treatment, and these miRNAs can be detected in both PB and BM samples. Notably, elevated levels of miR-665 were associated with treatment-refractory disease and correlated with poor survival outcomes and aggressive MM characteristics. These results highlight the potential role of non-invasive detection of miRNAs on drug response and survival outcomes in MM patients, paving the way for precision medicine. Furthermore, *in silico* analysis suggested that miR-665 target genes regulate cell proliferation and drug resistance, suggesting its possible role in MM heterogeneity and treatment resistance. Future research is needed to validate these results in larger cohorts and explore the underlying mechanisms of miR-665 in MM pathogenesis and drug resistance.

## Data Availability

Publicly available datasets were analyzed in this study. This data can be found here: https://www.ncbi.nlm.nih.gov/bioproject/1232635.

## References

[B1] Abd ElHafeez S. D'Arrigo G. Leonardis D. Fusaro M. Tripepi G. Roumeliotis S. (2021). Methods to analyze time-to-event data: the Cox regression analysis. Oxid. Med. Cell Longev. 2021, 1302811. 10.1155/2021/1302811 34887996 PMC8651375

[B2] Abdi J. Jian H. Chang H. (2016). Role of micro-RNAs in drug resistance of multiple myeloma. Oncotarget 7 (37), 60723–60735. 10.18632/oncotarget.11032 27494872 PMC5312415

[B3] Ahmad N. Haider S. Jagannathan S. Anaissie E. Driscoll J. J. (2014). MicroRNA theragnostics for the clinical management of multiple myeloma. Leukemia 28 (4), 732–738. 10.1038/leu.2013.262 24714346

[B4] Allegra A. Ettari R. Innao V. Bitto A. (2021a). Potential role of microRNAs in inducing drug resistance in patients with multiple myeloma. Cells 10 (2), 448. 10.3390/cells10020448 33672466 PMC7923438

[B5] Allegra A. Ettari R. Innao V. Bitto A. (2021b). Potential role of microRNAs in inducing drug resistance in patients with multiple myeloma. Cells 10 (2), 448. 10.3390/cells10020448 33672466 PMC7923438

[B6] Andrés-León E. Rojas A. M. (2019). miARma-Seq, a comprehensive pipeline for the simultaneous study and integration of miRNA and mRNA expression data. Methods. 152, 31–40. 10.1016/j.ymeth.2018.09.002 30253202

[B7] Azaman I. Chng W.-J. Mustafa N. De Mel S. (2023). Extracellular vesicles secreted from daratumumab resistant cells promote resistance and proliferation of daratumumab sensitive cells, possibly through the transfer of miRNA cargo. Blood 142, 1443. 10.1182/blood-2023-185134

[B8] Bai H. Chen B. (2020). BAG3 regulates multiple myeloma cell proliferation through FOXM1/Rb/E2F axis. Cancer Gene Ther. 27 (1-2), 108–111. 10.1038/s41417-019-0154-2 31801989

[B9] Bergantim R. Jorge J. Peixoto da Silva S. Alves R. Gonçalves A. C. Sarmento-Ribeiro A. B. (2024). “The role of MicroRNAs in mature B-cell neoplasias drug resistance,” in Reference module in biomedical Sciences. Elsevier.

[B10] Bergantim R. Peixoto da Silva S. Polónia B. Barbosa M. A. G. Albergaria A. Lima J. (2022). Detection of measurable residual disease biomarkers in extracellular vesicles from liquid biopsies of multiple myeloma patients-A proof of concept. Int. J. Mol. Sci. 23 (22), 13686. 10.3390/ijms232213686 36430163 PMC9690807

[B11] Besse L. Sedlarikova L. Kryukov F. Nekvindova J. Radova L. Slaby O. (2015). Circulating serum MicroRNA-130a as a novel putative marker of extramedullary myeloma. PLoS One 10 (9), e0137294. 10.1371/journal.pone.0137294 26389804 PMC4577078

[B12] Bonello F. Cani L. D'Agostino M. (2022). Risk stratification before and during treatment in newly diagnosed multiple myeloma: from clinical trials to the real-world setting. Front. Oncol. 12, 830922. 10.3389/fonc.2022.830922 35356221 PMC8959380

[B13] Campo S. Allegra A. D'Ascola A. Alonci A. Scuruchi M. Russo S. (2014). MiRNome expression is deregulated in the peripheral lymphoid compartment of multiple myeloma. Br. J. Haematol. 165 (6), 801–813. 10.1111/bjh.12828 24620752

[B14] Caracciolo D. Riillo C. Juli G. Scionti F. Todoerti K. Polerà N. (2021). miR-22 modulates lenalidomide activity by counteracting MYC addiction in multiple myeloma. Cancers (Basel) 13 (17), 4365. 10.3390/cancers13174365 34503175 PMC8431372

[B15] Carvajal-Vergara X. Tabera S. Montero J. C. Esparís-Ogando A. López-Pérez R. Mateo G. (2005). Multifunctional role of Erk5 in multiple myeloma. Blood 105 (11), 4492–4499. 10.1182/blood-2004-08-2985 15692064

[B16] Chen D. Yang X. Liu M. Zhang Z. Xing E. (2021). Roles of miRNA dysregulation in the pathogenesis of multiple myeloma. Cancer Gene Ther. 28 (12), 1256–1268. 10.1038/s41417-020-00291-4 33402729 PMC8636266

[B17] Chen Y. Wang X. (2020). miRDB: an online database for prediction of functional microRNA targets. Nucleic Acids Res. 48 (D1), D127-D131–D31. 10.1093/nar/gkz757 31504780 PMC6943051

[B18] Cheng L. Hill A. F. (2017). Small RNA library construction for exosomal RNA from biological samples for the Ion torrent PGM™ and Ion S5™ System. Methods Mol. Biol. 1545, 71–90. 10.1007/978-1-4939-6728-5_6 27943208

[B19] Corp I. (2023). IBM SPSS Statistics for windows, version 29.0.2.0. Armonk, NY: IBM Corp.

[B20] Damanti C. C. Gaffo E. Lovisa F. Garbin A. Di Battista P. Gallingani I. (2021). MiR-26a-5p as a reference to normalize MicroRNA qRT-PCR levels in plasma exosomes of pediatric hematological malignancies. Cells 10 (1), 101. 10.3390/cells10010101 33429910 PMC7827902

[B21] De Marco M. Turco M. C. Marzullo L. (2020). BAG3 in tumor resistance to therapy. Trends Cancer 6 (12), 985–988. 10.1016/j.trecan.2020.07.001 32718905

[B22] Diamond B. T. Rustad E. Maclachlan K. Thoren K. Ho C. Roshal M. (2021). Defining the undetectable: the current landscape of minimal residual disease assessment in multiple myeloma and goals for future clarity. Blood Rev. 46, 100732. 10.1016/j.blre.2020.100732 32771227 PMC9928431

[B23] Dimopoulos M. A. Moreau P. Terpos E. Mateos M. V. Zweegman S. Cook G. (2021). Multiple myeloma: EHA-ESMO Clinical Practice Guidelines for diagnosis, treatment and follow-up. Ann. Oncol. 32 (3), 309–322. 10.1016/j.annonc.2020.11.014 33549387

[B24] Durinck S. Spellman P. T. Birney E. Huber W. (2009). Mapping identifiers for the integration of genomic datasets with the R/Bioconductor package biomaRt. Nat. Protoc. 4 (8), 1184–1191. 10.1038/nprot.2009.97 19617889 PMC3159387

[B25] Egan J. B. Shi C. X. Tembe W. Christoforides A. Kurdoglu A. Sinari S. (2012). Whole-genome sequencing of multiple myeloma from diagnosis to plasma cell leukemia reveals genomic initiating events, evolution, and clonal tides. Blood 120 (5), 1060–1066. 10.1182/blood-2012-01-405977 22529291 PMC3412329

[B26] Fang T. Sun H. Sun X. He Y. Tang P. Gong L. (2023). Exosome miRNAs profiling in serum and prognostic evaluation in patients with multiple myeloma. Blood Sci. 5 (3), 196–208. 10.1097/BS9.0000000000000160 37546707 PMC10400059

[B27] Gengenbach L. Graziani G. Reinhardt H. Rösner A. Braun M. Möller M.-D. (2021). Choosing the right therapy for patients with relapsed/refractory multiple myeloma (RRMM) in consideration of patient-disease- and treatment-related factors. Cancers 13 (17), 4320. 10.3390/cancers13174320 34503130 PMC8430818

[B28] Giovannetti E. Erozenci A. Smit J. Danesi R. Peters G. J. (2012). Molecular mechanisms underlying the role of microRNAs (miRNAs) in anticancer drug resistance and implications for clinical practice. Crit. Rev. Oncol. Hematol. 81 (2), 103–122. 10.1016/j.critrevonc.2011.03.010 21546262

[B29] Gregorova J. Radova L. Gablo N. A. Almasi M. Stork M. Slaby O. (2019). MicroRNA analysis in multiple myeloma and extramedullary disease. Clin. Lymphoma, Myeloma Leukemia 19 (10), e65–e66. 10.1016/j.clml.2019.09.103

[B30] Gu J. Wang M. Wang X. Li J. Liu H. Lin Z. (2022). Exosomal miR-483-5p in bone marrow mesenchymal stem cells promotes malignant progression of multiple myeloma by targeting TIMP2. Front. Cell Dev. Biol. 10, 862524. 10.3389/fcell.2022.862524 35300408 PMC8921260

[B31] Guan X. Pavani K. C. Chunduru J. Broeckx B. J. G. Van Soom A. Peelman L. (2023). Hsa-miR-665 is a promising biomarker in cancer prognosis. Cancers (Basel). 15 (20), 4915. 10.3390/cancers15204915 37894282 PMC10605552

[B32] Gulla A. Anderson K. C. (2020). Multiple myeloma: the (r)evolution of current therapy and a glance into future. Haematologica 105 (10), 2358–2367. 10.3324/haematol.2020.247015 33054076 PMC7556665

[B33] Gutiérrez N. C. Sarasquete M. E. Misiewicz-Krzeminska I. Delgado M. De Las Rivas J. Ticona F. V. (2010). Deregulation of microRNA expression in the different genetic subtypes of multiple myeloma and correlation with gene expression profiling. Leukemia 24 (3), 629–637. 10.1038/leu.2009.274 20054351

[B34] Ha M. Kim V. N. (2014). Regulation of microRNA biogenesis. Nat. Rev. Mol. Cell Biol. 15 (8), 509–524. 10.1038/nrm3838 25027649

[B35] Handa H. Murakami Y. Ishihara R. Kimura-Masuda K. Masuda Y. (2019). The role and function of microRNA in the pathogenesis of multiple myeloma. Cancers (Basel) 11 (11), 1738. 10.3390/cancers11111738 31698726 PMC6896016

[B36] Harman J. R. Thorne R. Jamilly M. Tapia M. Crump N. T. Rice S. (2021). A KMT2A-AFF1 gene regulatory network highlights the role of core transcription factors and reveals the regulatory logic of key downstream target genes. Genome Res. 31 (7), 1159–1173. 10.1101/gr.268490.120 34088716 PMC8256865

[B37] Hernández-Rivas J.-Á. Ríos-Tamayo R. Encinas C. Alonso R. Lahuerta J.-J. (2022). The changing landscape of relapsed and/or refractory multiple myeloma (MM): fundamentals and controversies. Biomark. Res. 10 (1), 1. 10.1186/s40364-021-00344-2 35000618 PMC8743063

[B38] Höffken V. Hermann A. Pavenstädt H. Kremerskothen J. (2021). WWC proteins: important regulators of hippo signaling in cancer. Cancers (Basel) 13 (2), 306. 10.3390/cancers13020306 33467643 PMC7829927

[B39] Huang H. P. Liu W. J. Guo Q. L. Bai Y. Q. (2016). Effect of silencing HOXA5 gene expression using RNA interference on cell cycle and apoptosis in Jurkat cells. Int. J. Mol. Med. 37 (3), 669–678. 10.3892/ijmm.2016.2480 26846409 PMC4771120

[B40] Huang J. J. Yu J. Li J. Y. Liu Y. T. Zhong R. Q. (2012). Circulating microRNA expression is associated with genetic subtype and survival of multiple myeloma. Med. Oncol. 29 (4), 2402–2408. 10.1007/s12032-012-0210-3 22447484

[B41] Ismail N. H. Mussa A. Al-Khreisat M. J. Mohamed Y. S. Husin A. Han A.-J. (2023). Dysregulation of non-coding RNAs: roles of miRNAs and lncRNAs in the pathogenesis of multiple myeloma. Noncoding RNA 9 (6), 68. 10.3390/ncrna9060068 37987364 PMC10660696

[B42] Jagannathan S. Vad N. Vallabhapurapu S. Vallabhapurapu S. Anderson K. C. Driscoll J. J. (2015). MiR-29b replacement inhibits proteasomes and disrupts aggresome+autophagosome formation to enhance the antimyeloma benefit of bortezomib. Leukemia 29 (3), 727–738. 10.1038/leu.2014.279 25234165 PMC4360212

[B43] Jurczyszyn A. Waszczuk-Gajda A. Castillo J. J. Krawczyk K. Stork M. Pour L. (2020). Primary refractory multiple myeloma: a real-world experience with 85 cases. Leuk. Lymphoma 61 (12), 2868–2875. 10.1080/10428194.2020.1788014 32623944

[B44] Krishnan S. R. Bebawy M. (2023). Circulating biosignatures in multiple myeloma and their role in multidrug resistance. Mol. Cancer 22 (1), 79. 10.1186/s12943-022-01683-w 37120508 PMC10148481

[B45] Kubiczkova L. Kryukov F. Slaby O. Dementyeva E. Jarkovsky J. Nekvindova J. (2014). Circulating serum microRNAs as novel diagnostic and prognostic biomarkers for multiple myeloma and monoclonal gammopathy of undetermined significance. Haematologica 99 (3), 511–518. 10.3324/haematol.2013.093500 24241494 PMC3943315

[B46] Kuleshov M. V. Jones M. R. Rouillard A. D. Fernandez N. F. Duan Q. Wang Z. (2016). Enrichr: a comprehensive gene set enrichment analysis web server 2016 update. Nucleic Acids Res. 44 (W1), W90–W97. 10.1093/nar/gkw377 27141961 PMC4987924

[B47] Kumar S. Paiva B. Anderson K. C. Durie B. Landgren O. Moreau P. (2016). International Myeloma Working Group consensus criteria for response and minimal residual disease assessment in multiple myeloma. Lancet Oncol. 17 (8), e328–e346. 10.1016/S1470-2045(16)30206-6 27511158

[B48] Lahuerta J. J. Paiva B. Jiménez de Ubieto A. Sánchez-Pina J. Mateos M.-V. Bladé J. (2021). Early detection of treatment failure and early rescue intervention in multiple myeloma: time for new approaches. Blood Adv. 5 (5), 1340–1343. 10.1182/bloodadvances.2020003996 33656540 PMC7948301

[B49] Leung-Hagesteijn C. Erdmann N. Cheung G. Keats J. J. Stewart A. K. Reece D. E. (2013). Xbp1s-negative tumor B cells and pre-plasmablasts mediate therapeutic proteasome inhibitor resistance in multiple myeloma. Cancer Cell 24 (3), 289–304. 10.1016/j.ccr.2013.08.009 24029229 PMC4118579

[B50] Li J. Wang C. Meng Q. Hu Z. Hu M. Zhang M. (2021). MicroRNAs in urine as diagnostic biomarkers for multiple myeloma. Int. J. Laboratory Hematol. 43 (2), 227–234. 10.1111/ijlh.13367 33068078

[B51] Liao Y. Smyth G. K. Shi W. (2014). featureCounts: an efficient general purpose program for assigning sequence reads to genomic features. Bioinformatics 30 (7), 923–930. 10.1093/bioinformatics/btt656 24227677

[B52] Lin S. Gregory R. I. (2015). MicroRNA biogenesis pathways in cancer. Nat. Rev. Cancer 15 (6), 321–333. 10.1038/nrc3932 25998712 PMC4859809

[B53] Liu N. W. Huang X. Liu S. Lu Y. (2019). EXT1, regulated by MiR-665, promotes cell apoptosis via ERK1/2 signaling pathway in acute lymphoblastic leukemia. Med. Sci. Monit. 25, 6491–6503. 10.12659/MSM.918295 31465316 PMC6733154

[B54] Liu Y. Kywe B. Crawford L. Lora F. Bar N. Browning S. L. (2021). Outcomes among primary refractory multiple myeloma patients in the era of monoclonal antibodies: the yale experience. Blood 138 (Suppl. 1), 1635. 10.1182/blood-2021-146619

[B55] Liu Y. Li H. Zhao Y. Li D. Zhang Q. Fu J. (2022). Knockdown of ADORA2A antisense RNA 1 inhibits cell proliferation and enhances imatinib sensitivity in chronic myeloid leukemia. Bioengineered 13 (2), 2296–2307. 10.1080/21655979.2021.2024389 35034552 PMC8973732

[B56] Liu Z. Xu J. He J. Zheng Y. Li H. Lu Y. (2014). A critical role of autocrine sonic hedgehog signaling in human CD138+ myeloma cell survival and drug resistance. Blood 124 (13), 2061–2071. 10.1182/blood-2014-03-557298 25049282 PMC4186536

[B57] Majithia N. Vincent Rajkumar S. Lacy M. Q. Buadi F. K. Dispenzieri A. Gertz M. A. (2015). Outcomes of primary refractory multiple myeloma and the impact of novel therapies. Am. J. Hematol. 90 (11), 981–985. 10.1002/ajh.24131 26214732 PMC5801545

[B58] McGeary S. E. Lin K. S. Shi C. Y. Pham T. M. Bisaria N. Kelley G. M. (2019). The biochemical basis of microRNA targeting efficacy. Science. 366 (6472), eaav1741. 10.1126/science.aav1741 31806698 PMC7051167

[B59] Miao Y. Medeiros L. J. Li Y. Li J. Young K. H. (2019). Genetic alterations and their clinical implications in DLBCL. Nat. Rev. Clin. Oncol. 16 (10), 634–652. 10.1038/s41571-019-0225-1 31127191

[B60] Moreau P. Kumar S. K. Miguel J. S. Davies F. Zamagni E. Bahlis N. (2021). Treatment of relapsed and refractory multiple myeloma: recommendations from the International Myeloma Working Group. Lancet Oncol. 22 (3), e105–e118. 10.1016/S1470-2045(20)30756-7 33662288

[B61] Munshi N. C. Avet-Loiseau H. Anderson K. C. Neri P. Paiva B. Samur M. (2020). A large meta-analysis establishes the role of MRD negativity in long-term survival outcomes in patients with multiple myeloma. Blood Adv. 4 (23), 5988–5999. 10.1182/bloodadvances.2020002827 33284948 PMC7724898

[B62] Muylaert C. Van Hemelrijck L. A. Maes A. De Veirman K. Menu E. Vanderkerken K. (2022). Aberrant DNA methylation in multiple myeloma: a major obstacle or an opportunity? Front. Oncol. 12, 979569. 10.3389/fonc.2022.979569 36059621 PMC9434119

[B63] Niture S. Gadi S. Qi Q. Gyamfi M. A. Varghese R. S. Rios-Colon L. (2023). MicroRNA-483-5p inhibits hepatocellular carcinoma cell proliferation, cell steatosis, and fibrosis by targeting PPARα and TIMP2. Cancers (Basel) 15 (6), 1715. 10.3390/cancers15061715 36980601 PMC10046356

[B64] Ohata Y. Chirgwin J. M. Windle J. J. Roodman G. D. Kurihara N. (2016). Myeloma cells induce high level of TAF12 expression in bone marrow stromal cells, resulting in increased osteoclastogenesis and myeloma cell growth in response to 1,25(OH)2D3. Blood 128 (22), 4421. 10.1182/blood.v128.22.4421.4421

[B65] Paraskevopoulou M. D. Georgakilas G. Kostoulas N. Vlachos I. S. Vergoulis T. Reczko M. (2013). DIANA-microT web server v5.0: service integration into miRNA functional analysis workflows. Nucleic Acids Res. 41 (Web Server issue), W169–W173. 10.1093/nar/gkt393 23680784 PMC3692048

[B66] Pasca S. Tomuleasa C. Teodorescu P. Ghiaur G. Dima D. Moisoiu V. (2019). KRAS/NRAS/BRAF mutations as potential targets in multiple myeloma. Front. Oncol. 9, 1137. 10.3389/fonc.2019.01137 31709194 PMC6821642

[B67] Pedroza-Torres A. Romero-Córdoba S. L. Justo-Garrido M. Salido-Guadarrama I. Rodríguez-Bautista R. Montaño S. (2019). MicroRNAs in tumor cell metabolism: roles and therapeutic opportunities. Front. Oncol. 9, 1404. 10.3389/fonc.2019.01404 31921661 PMC6917641

[B68] Peixoto da Silva S. Caires H. R. Bergantim R. Guimarães J. E. Vasconcelos M. H. (2021). miRNAs mediated drug resistance in hematological malignancies. Semin. Cancer Biol. 83, 283–302. 10.1016/j.semcancer.2021.03.014 33757848

[B69] Perroud C. Thurian D. Andres M. Künzi A. Wiedemann G. Zeerleder S. (2023). Effect of MAPK activation via mutations in NRAS, KRAS and BRAF on clinical outcome in newly diagnosed multiple myeloma. Hematol. Oncol. 41 (5), 912–921. 10.1002/hon.3208 37452600

[B70] Pfaffl M. W. Tichopad A. Prgomet C. Neuvians T. P. (2004). Determination of stable housekeeping genes, differentially regulated target genes and sample integrity: BestKeeper--Excel-based tool using pair-wise correlations. Biotechnol. Lett. 26 (6), 509–515. 10.1023/b:bile.0000019559.84305.47 15127793

[B71] Pinto V. Bergantim R. Caires H. R. Seca H. Guimarães J. E. Vasconcelos M. H. (2020). Multiple myeloma: available therapies and causes of drug resistance. Cancers (Basel) 12 (2), 407. 10.3390/cancers12020407 32050631 PMC7072128

[B72] Pomaznoy M. Ha B. Peters B. (2018). GOnet: a tool for interactive Gene Ontology analysis. BMC Bioinforma. 19 (1), 470. 10.1186/s12859-018-2533-3 PMC6286514 30526489

[B73] Prism G. (2024). GraphPad prism, version 10.0.0 for mac. Boston, Massachusetts USA: GraphPad Software.

[B74] Qu X. Zhao M. Wu S. Yu W. Xu J. Xu J. (2014). Circulating microRNA 483-5p as a novel biomarker for diagnosis survival prediction in multiple myeloma. Med. Oncol. 31 (10), 219. 10.1007/s12032-014-0219-x 25216866

[B75] Rajkumar S. V. Dimopoulos M. A. Palumbo A. Blade J. Merlini G. Mateos M. V. (2014). International Myeloma Working Group updated criteria for the diagnosis of multiple myeloma. Lancet Oncol. 15 (12), e538–e548. 10.1016/S1470-2045(14)70442-5 25439696

[B76] Rajkumar S. V. Harousseau J. L. Durie B. Anderson K. C. Dimopoulos M. Kyle R. (2011). Consensus recommendations for the uniform reporting of clinical trials: report of the International Myeloma Workshop Consensus Panel 1. Blood 117 (18), 4691–4695. 10.1182/blood-2010-10-299487 21292775 PMC3710442

[B77] Rattanapan Y. Korkiatsakul V. Kongruang A. Siriboonpiputtana T. Rerkamnuaychoke B. Chareonsirisuthigul T. (2021). High expression of miR-483-5p predicts chemotherapy resistance in epithelial ovarian cancer. Microrna 10 (1), 51–57. 10.2174/2211536610666210412155206 33845755

[B78] Robinson M. D. McCarthy D. J. Smyth G. K. (2010). edgeR: a Bioconductor package for differential expression analysis of digital gene expression data. Bioinformatics 26 (1), 139–140. 10.1093/bioinformatics/btp616 19910308 PMC2796818

[B79] Rocci A. Hofmeister C. C. Geyer S. Stiff A. Gambella M. Cascione L. (2014a). Circulating miRNA markers show promise as new prognosticators for multiple myeloma. Leukemia 28 (9), 1922–1926. 10.1038/leu.2014.155 24813918 PMC4155011

[B80] Rocci A. Hofmeister C. C. Pichiorri F. (2014b). The potential of miRNAs as biomarkers for multiple myeloma. Expert Rev. Mol. Diagn 14 (8), 947–959. 10.1586/14737159.2014.946906 25098410

[B81] Rodriguez-Otero P. Paiva B. San-Miguel J. F. (2021). Roadmap to cure multiple myeloma. Cancer Treat. Rev. 100, 102284. 10.1016/j.ctrv.2021.102284 34597912

[B82] Schmieder R. Edwards R. (2011). Quality control and preprocessing of metagenomic datasets. Bioinformatics 27 (6), 863–864. 10.1093/bioinformatics/btr026 21278185 PMC3051327

[B83] Sha Z. Schnell H. M. Ruoff K. Goldberg A. (2018). Rapid induction of p62 and GABARAPL1 upon proteasome inhibition promotes survival before autophagy activation. J. Cell Biol. 217 (5), 1757–1776. 10.1083/jcb.201708168 29535191 PMC5940303

[B84] Sousa D. Matthiesen R. Lima R. T. Vasconcelos M. H. (2020). Deep sequencing analysis reveals distinctive non-coding RNAs when comparing tumor multidrug-resistant cells and extracellular vesicles with drug-sensitive counterparts. Cancers (Basel) 12 (1), 200. 10.3390/cancers12010200 31947507 PMC7016831

[B85] Suzuki H. I. Katsura A. Matsuyama H. Miyazono K. (2015). MicroRNA regulons in tumor microenvironment. Oncogene 34 (24), 3085–3094. 10.1038/onc.2014.254 25132266 PMC4761641

[B86] Thorsteinsdottir S. Dickman P. W. Landgren O. Blimark C. Hultcrantz M. Turesson I. (2018). Dramatically improved survival in multiple myeloma patients in the recent decade: results from a Swedish population-based study. Haematologica 103 (9), e412–e415. 10.3324/haematol.2017.183475 29567776 PMC6119139

[B87] Tian Y. Yan M. Zheng J. Li R. Lin J. Xu A. (2019). miR-483-5p decreases the radiosensitivity of nasopharyngeal carcinoma cells by targeting DAPK1. Lab. Invest 99 (5), 602–611. 10.1038/s41374-018-0169-6 30664712

[B88] Wang F. Zhang X. Zhong X. Zhang M. Guo M. Yang L. (2018). Effect of miR-483-5p on apoptosis of lung cancer cells through targeting of RBM5. Int. J. Clin. Exp. Pathol. 11 (6), 3147–3156. 31938444 PMC6958086

[B89] Wang Y. Guo D. Li B. Wang Y. Wang B. Wang Z. (2022). MiR-665 suppresses the progression of diffuse large B cell lymphoma (DLBCL) through targeting LIM and SH3 protein 1 (LASP1). Leuk. Res. 112, 106769. 10.1016/j.leukres.2021.106769 34875555

[B90] Wu K. Z. Zhang C. D. Zhang C. Pei J. P. Dai D. Q. (2020). miR-665 suppresses the epithelial-mesenchymal transition and progression of gastric cancer by targeting CRIM1. Cancer Manag. Res. 12, 3489–3501. 10.2147/CMAR.S241795 32523379 PMC7237120

[B91] Xiang Y. Zhang L. Xiang P. Zhang J. (2021). Circulating miRNAs as auxiliary diagnostic biomarkers for multiple myeloma: a systematic review, meta-analysis, and recommendations. Front. Oncol. 11. 10.3389/fonc.2021.698197 PMC8297545 34307166

[B92] Xie F. Wang J. Zhang B. (2023). RefFinder: a web-based tool for comprehensively analyzing and identifying reference genes. Funct. Integr. Genomics 23 (2), 125. 10.1007/s10142-023-01055-7 37060478

[B93] Yang S. Zhang K. Fang Z. (2022). Robust RNA-seq data analysis using an integrated method of ROC curve and Kolmogorov-Smirnov test. Commun. Stat. Simul. Comput. 51 (12), 7444–7457. 10.1080/03610918.2020.1837165 36583130 PMC9793859

[B94] Yang Z. G. Ma X. D. He Z. H. Guo Y. X. (2017). miR-483-5p promotes prostate cancer cell proliferation and invasion by targeting RBM5. Int. Braz J. Urol. 43 (6), 1060–1067. 10.1590/S1677-5538.IBJU.2016.0595 28727371 PMC5734068

[B95] Yazgili A. S. Ebstein F. Meiners S. (2022). The proteasome activator pa200/PSME4: an emerging new player in Health and disease. Biomolecules 12 (8), 1150. 10.3390/biom12081150 36009043 PMC9406137

[B96] Zhang B. Pan X. Cobb G. P. Anderson T. A. (2007). microRNAs as oncogenes and tumor suppressors. Dev. Biol. 302 (1), 1–12. 10.1016/j.ydbio.2006.08.028 16989803

[B97] Zhang J. Xiao X. Liu J. (2015). The role of circulating miRNAs in multiple myeloma. Sci. China Life Sci. 58 (12), 1262–1269. 10.1007/s11427-015-4969-2 26607481

[B98] Zhang L. Pan L. Xiang B. Zhu H. Wu Y. Chen M. (2016). Potential role of exosome-associated microRNA panels and *in vivo* environment to predict drug resistance for patients with multiple myeloma. Oncotarget 7 (21), 30876–30891. 10.18632/oncotarget.9021 27129167 PMC5058725

[B99] Zhang W. Li Q. Zhang Y. Wang Z. Yuan S. Zhang X. (2023). Multiple myeloma with high expression of SLC7A11 is sensitive to erastin-induced ferroptosis. Apoptosis. 29, 412, 423. 10.1007/s10495-023-01909-2 38001343

[B100] Zhu B. Ju S. Chu H. Shen X. Zhang Y. Luo X. (2018). The potential function of microRNAs as biomarkers and therapeutic targets in multiple myeloma. Oncol. Lett. 15 (5), 6094–6106. 10.3892/ol.2018.8157 29731841 PMC5920744

[B101] Zub K. A. Sousa M. M. Sarno A. Sharma A. Demirovic A. Rao S. (2015). Modulation of cell metabolic pathways and oxidative stress signaling contribute to acquired melphalan resistance in multiple myeloma cells. PLoS One 10 (3), e0119857. 10.1371/journal.pone.0119857 25769101 PMC4358942

[B102] Zuo X. Liu D. (2022). Mechanism of immunomodulatory drug resistance and novel therapeutic strategies in multiple myeloma. Hematology 27 (1), 1110–1121. 10.1080/16078454.2022.2124694 36121114

