# Supplementary material for: MicroRNA-665 and its potential role in drug response and survival outcomes in multiple myeloma: a preliminary study
Source: Front Pharmacol. 2025 Apr 4;16:1465814. doi: 10.3389/fphar.2025.1465814 (PMC12006192; doi:10.3389/fphar.2025.1465814)
Supplement: Supplementary file 1 [file DataSheet1.docx]

Supplementary Material

*
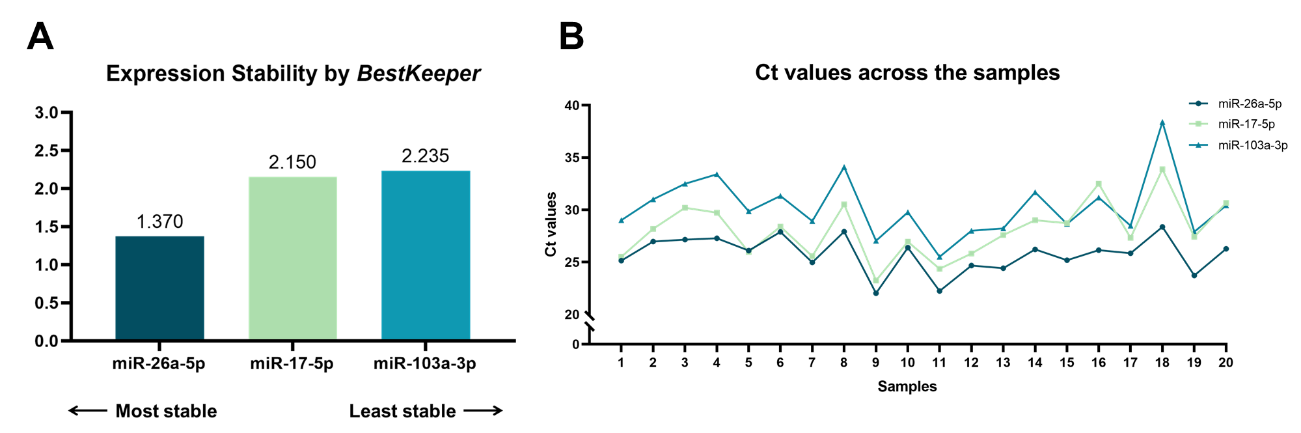
*

Supplementary Figure 1. Evaluation of the candidates to use as endogenous normalizer. A) Stability analyses of the expression of miRNAs normalizer candidates by BestKeeper software. This analysis indicates that miR-26a-5p is the most stable among all samples from the different groups. B) Cycle threshold (Ct) values across all samples of the different groups for the three candidate endogenous normalizers. The miR-26a-5p presents the lower Ct values and the most stable expression in all the samples from the different groups compared to the other two candidates.

**Supplementary Figure 2.** ROC curve analysis of miR-665 levels for the discrimination of high and low expression in MM samples from refractory and sensitive patients. The optimal cut-off for miR-665, determined by Youden’s index, was ΔCT 0.2206, with a sensitivity of 100% and a specificity of 33%. AUC: area under the curve, 95% Cl: 95% confidence intervals. *p* value calculated by the Hanley and McNeil method.

**C
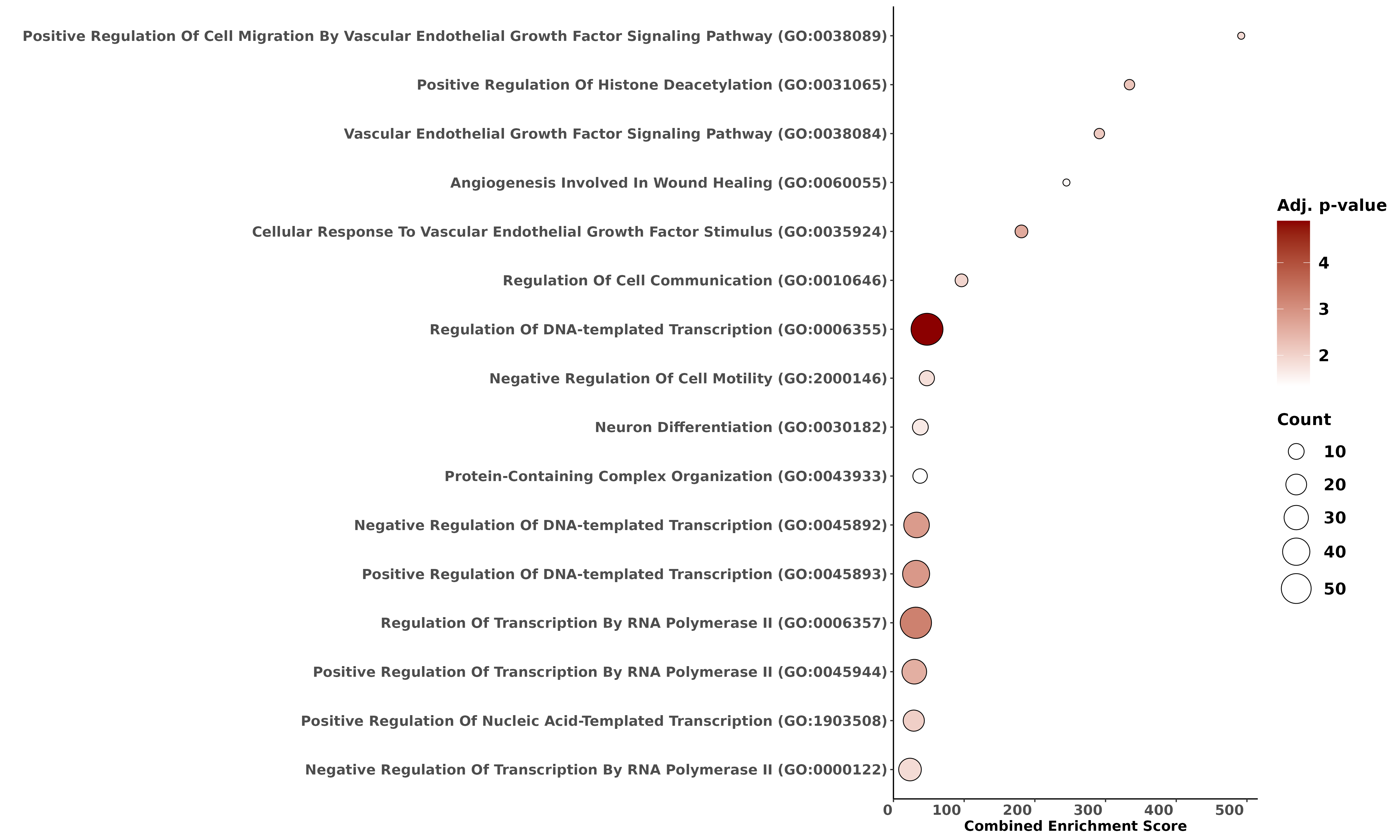
**

**Supplementary Figure 3.** Gene Ontology (GO) enrichment analysis of miR‐665. **A)** Venn diagram shows the number of significant GO terms according to miRDB, TargetScanHuman 8.0, and DIANA TOOLS-microT- CDS target prediction tools. **B)** Significant GO terms associated with miR-665 target genes: the vertical axis indicates the combined enrichment score, while the horizontal axis categorizes the GO terms. **C)** Dot plot showing enrichment of GO biological processes. The color of each dot corresponds to the *p*-value, and the size indicates the number of miR-665 target genes associated with each biological process term.

**Supplementary Table 1.** Baseline and clinical characteristics of Multiple Myeloma (MM) patients according to their expression of miR-665.

| **Baseline and Clinical Characteristics** | **Total (n=20)** | **High miR-665 expression** | **Low miR-665 expression**  **low** | ***p*-value** |
| --- | --- | --- | --- | --- |
|  |  |  |  |  |
| **Age** |  |  |  |  |
| **Median (range) - years** | 58 (44-67) | 56 (48-63) | 60 (44-67%) | 0.589 |
| **Sex - no. (%)** |  |  |  |  |
| Male | 12 (60%) | 7 (35%) | 5 (25%) | **0.040*** |
| Female | 8 (40%) | 1 (5%) | 7 (35%) |  |
| **Myeloma isotype - no (%)** |  |  |  |  |
| Light Chain Kappa | 4 (20%) | 2 (10%) | 2 (10%) | 0.645 |
| IgG/k | 6 (30%) | 1 (5%) | 5 (25%) |  |
| IgG/L | 5 (25%) | 2 (10%) | 3 (15%) |  |
| IgA/k | 3 (15%) | 2 (10%) | 1 (5%) |  |
| IgA/L | 2 (10%) | 1 (5%) | 1 (5%) |  |
| **ISS stage - no (%)** |  |  |  |  |
| I | 3 (15%) | 1 (5%) | 2 (10%) | 0.073 |
| II | 8 (40%) | 1 (5%) | 7 (35%) |  |
| III | 9 (45%) | 6 (30%) | 3 (15%) |  |
| **R-ISS stage - no (%)** |  |  |  |  |
| I | 3 (15%) | 1 (5%) | 2 (10%) | **0.031*** |
| II | 11 (55%) | 2 (10%) | 9 (45%) |  |
| III | 6 (30%) | 5 (25%) | 1 (5%) |  |
| **FISH** |  |  |  |  |
| Normal | 11 (55%) | 3 (15%) | 8 (40%) | 0.199 |
| Abnormal | 9 (45%) | 5 (25%) | 4 (20%) |  |
| **High-risk cytogenetic profile** |  |  |  |  |
| del(17p) | 2 (22.2%) | 2 (22.2%) | 0 (0%) | 0.151 |
| t(4:14) | 2 (22.2%) | 2 (22.2%) | 0 (0%) | 0.151 |
| t(14;16) | 1 (11.1%) | 0 (0%) | 1 (11.1%) | 0.236 |
| t(11;14) | 3 (33.3%) | 1 (11.1%) | 2 (22.2%) | 0.343 |
| amp1q21 | 3 (33.3%) | 2 (22.2%) | 1 (11.1%) | 0.635 |
| Double-Hit (del17p and t(4;14)) | 2 (10%) | 2 (10%) | 0 (0%) | 0.151 |
| **Anemia** |  |  |  |  |
| <10g/dL or <1g/dL of normal Hb | 16 (80%) | 7 (35%) | 9 (45%) | 0.494 |
| **Creatinine** |  |  |  |  |
| <2mg/dl | 14 (70%) | 3 (15%) | 11 (55%) | **0.010*** |
| >=2mg/dL | 6 (30%) | 5 (25%) | 1 (5%) |  |
| **B2-microglobulin** |  |  |  |  |
| Increased | 9 (45%) | 6 (30%) | 3 (15%) | **0.028*** |
| Normal | 11 (55%) | 2 (10%) | 9 (45%) |  |
| **LDH** |  |  |  |  |
| Increased | 5 (25%) | 4 (20%) | 1 (5%) | **0.035*** |
| Normal | 15 (75%) | 4 (20%) | 11 (55%) |  |
| **Extramedullary plasmacytoma** |  |  |  |  |
| Yes | 3 (15%) | 3 (15%) | 0 (0%) | **0.021*** |
| No | 17 (85%) | 5 (25%) | 12 (60%) |  |
| **Clonal plasma cell in BM(median, range)** | 20.9 (5-72%) | 26.5 (5-66%) | 16.4 (5-72%) | 0.787 |
| **Clonal plasma cell in PB (median, range)** | 0 (0.35-4%) | 0 (0-1%) | 0 (0-4%) | 0.166 |

BM: bone marrow; FISH: Fluorescence in situ hybridization; ISS: International Staging System; LDH: Lactate dehydrogenase; PB: peripheral blood; R-ISS: Revised International Staging System; * *p* ≤0.05.

**Supplementary Table 2.** Univariate and multivariate Cox regression analyses regarding overall survival and progression-free survival of MM patients.

|  | **OVERALL SURVIVAL** | | | | | | **PROGRESSION FREE SURVIVAL** | | | | | |  |
| --- | --- | --- | --- | --- | --- | --- | --- | --- | --- | --- | --- | --- | --- |
|  | ***Univariate analysis OS*** | | | ***Multivariate analysis OS*** | | | ***Univariate analysis PFS*** | | | ***Multivariate analysis PFS*** | | |  |
| **n=20** |  |  |  |  |  |  |  |  |  |  |  |  |  |
| **Covariant** | **HR** | **95% CI** | ***p*-value** | **HR** | **95% CI** | ***p*-value** | **HR** | **95% CI** | ***p*-value** | **HR** | **95% CI** | ***p*-value** |  |
|  |  |  |  |  |  |  |  |  |  |  |  |  |  |
| **Circulating miR-665** |  |  |  |  |  |  |  |  |  |  |  |  |  |
|  | 1.787 | 1.145 – 2.789 | **0.011*** | 1.486 | 0.880-2.509 | 0.138 | 1.165 | 0.927-1.465 | 0.190 |  | n.d. |  |  |
| **R-ISS Stage** |  |  |  |  |  |  |  |  |  |  |  |  |  |
| R-ISS I/II (n=14) | 1 |  |  |  |  |  | 1 |  |  |  |  |  |  |
| R-ISS III (n=6) | 4.624 | 1.256-17.019 | **0.021*** | 0.486 | 0.051-4.651 | 0.531 | 1.376 | 0.440-4.302 | 0.583 |  | n.d. |  |  |
| **High-risk Cytogenetics** |  |  |  |  |  |  |  |  |  |  |  |  |  |
| No (n=5) | 1 |  |  |  | n.d. |  | 1 |  |  |  |  |  |  |
| Yes (n=4) | 0.547 | 0.118-2.533 | 0.441 |  |  |  | 0.776 | 0.193-3.121 | 0.721 |  | n.d. |  |  |
| **LDH** |  |  |  |  |  |  |  |  |  |  |  |  |  |
| ≤ 220 U/L (n=15) | 1 |  |  |  |  |  | 1 |  |  |  |  |  |  |
| ≥ 220 U/L (n=5) | 3.592 | 1.017-12.685 | **0.047*** | 1.772 | 0.150-20.949 | 0.650 | 1.311 | 0.415-4.143 | 0.645 |  | n.d. |  |  |
| **B2-microglobulin** |  |  |  |  |  |  |  |  |  |  |  |  |  |
| ≤ 5.5 mg/L (n=11) | 1 |  |  |  |  |  | 1 |  |  |  |  |  |  |
| ≥ 5.5 mg/L (n=9) | 2.826 | 0.815-9.808 | 0.102 |  | n.d. |  | 1.179 | 0.380-3.664 | 0.766 |  | n.d. |  |  |
| **Creatinine** |  |  |  |  |  |  |  |  |  |  |  |  |  |
| ≤ 2 mg/dL (n=14) | 1 |  |  |  | n.d. |  | 1 |  |  |  |  |  |  |
| ≥ 2 mg/dL (n=6) | 2.522 | 0.725-8.773 | 0.146 |  |  |  | 1.595 | 0.483-5.265 | 0.443 |  | n.d. |  |  |
| **EMD** |  |  |  |  |  |  |  |  |  |  |  |  |  |
| No (n=17) | 1 |  |  |  |  |  | 1 |  |  |  |  |  |  |
| Yes (n=3) | 0.228 | 0.05-1.37 | 0.056 |  | n.d. |  | 0.743 | 0.201-2.750 | 0.656 |  | n.d. |  |  |
| **Anemia** |  |  |  |  |  |  |  |  |  |  |  |  |  |
| No (n=4) | 1 |  |  |  |  |  | 1 |  |  |  |  |  |  |
| Yes (n=16) | 0.401 | 0.051-3.173 | 0.387 |  | n.d. |  | 1 | 0.000-2584 | 1 |  | n.d. |  |  |
| **Gender** |  |  |  |  |  |  |  |  |  |  |  |  |  |
| Female (n=8) | 1 |  |  |  |  |  | 1 |  |  |  |  |  |  |
| Male (n=12) | 0.171 | 0.036-0.812 | **0.026*** | 0.476 | 0.190-1.192 | 0.113 | 0.445 | 0.120-1.651 | 0.226 |  |  |  |  |
| **Age** |  |  |  |  |  |  |  |  |  |  |  |  |  |
|  | 0.979 | 0.901-1.063 | 0.607 |  | n.d. |  | 0.979 | 0.901-1.063 | 0.607 |  | n.d. |  |  |

EMD: Extramedullary disease; FISH: Fluorescence in situ hybridization; HR: hazard-ratio; ISS: International Staging System; LDH: Lactate dehydrogenase; miR: microRNA; OS: overall survival; PFS: progression-free survival; R-ISS: Revised International Staging System; 95% CI: 95% confidence interval of the estimated HR; n.d. not done, once no statistical significance was found on univariate analysis; * *p* ≤0.05.
